# Impact of the adenosine receptor A2BR expressed on myeloid cells on immune regulation during pregnancy

**DOI:** 10.1002/eji.202451149

**Published:** 2024-10-25

**Authors:** Stefanie Dietz, Janine Hebel, Jessica Rühle, Alisha Huff, Holger K. Eltzschig, Trim Lajqi, Christian F. Poets, Christian Gille, Natascha Köstlin‐Gille

**Affiliations:** ^1^ Department of Neonatology Tuebingen University Children's Hospital Tuebingen Germany; ^2^ Department of Neonatology Heidelberg University, Medical Faculty Heidelberg Germany; ^3^ Health Science Center Houston University of Texas Houston Texas USA

**Keywords:** Myeloid A2BR knockout, Adenosine receptor, Immune regulation, Pregnancy

## Abstract

During pregnancy, the maternal immune system must carefully balance protection against pathogens with tolerance toward the semiallogeneic fetus. Dysfunctions of the immune system can lead to severe complications such as preeclampsia, fetal growth restriction, or pregnancy loss. Adenosine plays a role in physiological processes and plasma‐level increase during pregnancy. The adenosine receptor A2B (A2BR), which is expressed on both, immune and nonimmune cells, is activated by high adenosine concentrations, achieved during pregnancy. We investigated the impact of A2BR expressed on myeloid cells on immune regulation during pregnancy using a mouse model with myeloid deficiency for A2BR. We demonstrate systemic changes in myeloid and lymphoid cell populations during pregnancy in A2BR‐KO (Adora2B923^f/f^‐LysM^Cre^) mice with increased monocytes, neutrophils, and T cells but decreased B cells as well as altered T‐cell subpopulations with decreased Th1 cells and Tregs and increased Th17 cells. Lack of A2BR on myeloid cells caused an increased systemic expression of IL‐6 but decreased systemic accumulation and function of MDSC and reduced numbers of uterine natural killer cells. The pregnancy outcome was only marginally affected. Our results demonstrate that A2BR on myeloid cells plays a role in immune regulation during pregnancy, but the clinical impact on pregnancy remains unclear.

## Graphical abstract

The absence of adenosine receptor A2B on myeloid cells in Adora2B923^f/f^‐LysM^Cre^ mice resulted in systemic changes in myeloid and lymphoid cell populations during pregnancy. There was an increased expression of IL‐6 and a reduction of MDSC and uNK cells. However, there were only marginal differences in pregnancy outcomes.

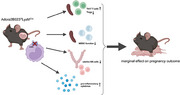

## Introduction

Abortions are one of the most common pregnancy complications. Approximately 15% of clinically recognized pregnancies result in miscarriage, while total reproductive losses are estimated to be closer to 50% [1]. Preterm birth is defined as birth before 37 weeks of gestation and accounts for 75% of perinatal mortality and morbidity [2]. One of the leading maternal causes of preterm birth is preeclampsia (PE), which is a hypertensive pregnancy disorder, characterized by elevated blood pressure (>140/90 mmHg) and proteinuria (>300 mg/day) in the second half of pregnancy [3]. The causes of abortion, preterm birth, and PE are diverse and incompletely understood. However, a dysregulation of the immune system in this special period of life appears to play an important role. During pregnancy, there is a close interaction between fetal cells and the mother's immune system [4, 5] and the maternal immune system has to be tightly regulated to prevent rejection of the fetus. Failure of the immunological adaptation processes to pregnancy can result in the development of pregnancy complications, including abortions and preterm birth.

For a long time, pregnancy was considered an immunological phenomenon mainly driven by a change in the T‐cell response, in particular a shift in the T helper cell response toward T helper 2 and an induction of regulatory T cells [6]. It is now evident that the functions of numerous other immune cell types are also altered during pregnancy. For example, in contrast to peripheral natural killer (NK) cells, uterine NK cells are not cytotoxic, but contribute to the structural remodeling of the spiral arteries and implantation of the embryo [7]. Another cell type that has been identified as playing a crucial role in a successful pregnancy is the so‐called myeloid‐derived suppressor cells (MDSC) [8, 9, 10, 11, 12, 13]. These cells are myeloid cells with suppressive activity on other immune cells, particularly on T cells, modulating immunity toward tolerance [6, 14].

Adenosine triphosphate is an intracellular energy source, but it can also be released to the extracellular environment, where it serves as a danger‐associated molecular pattern for the immune system [15, 16, 17]. An ATP release occurs in response to stress, cell damage, and necrosis, as well as in a regulated manner via exocytosis or through transmembrane channels [16, 18, 19]. Extracellular ATP binds to purinergic P2 receptors on various immune cells [17] and tissues [20]. In endothelial cells, engagement of P2 receptors leads to activation and vasoconstriction; in immune cells, it induces activation of the NLRP3 inflammasome and other proinflammatory pathways [15]. Due to these effects, the extracellular concentrations of ATP must be regulated by enzymatic digestion which is mediated by the ectonucleotidases triphosphate di‐phosphohydrolase I (CD39) and ecto‐5′‐nucleotidase (CD73), driving the production of adenosine [15]. Adenosine binds to purinergic P1 adenosine receptors A1, A2A, A2B, and A3 [21], counteracting the effects of ATP and leading to vasodilatation and anti‐inflammatory effects in immune cells [16]. A1, A2A, and A3 are high‐affinity receptors for adenosine and get activated at physiological concentrations of adenosine, while A2B has a low affinity and is only activated by high adenosine concentrations, achieved in tissues that experience ischemia, trauma, or inflammation [22] and during pregnancy [23]. It is postulated that these receptors contribute to hemodynamic changes and angiogenesis during pregnancy [16].

A2BR is expressed in both, immune and nonimmune cells [22]. A2BR expression is regulated by several factors, including metabolomic, inflammatory, or hormonal changes as well as by adenosine itself. Especially macrophages and endothelial cells exhibit high levels of A2BR expression [24]. Previous studies have indicated that mast cells [25], lymphocytes [26, 27], dendritic cells [28, 29], and neutrophils [27] also express A2BR. Under tumor conditions, A2BR has been described to promote tumor growth and tumor metastasis [30, 31], while in the context of inflammatory conditions, it dampens immune activation [32, 33, 34].

Based on the observations of A2BR under tumor conditions and the fact that significantly increased levels of adenosine are present during pregnancy, we aimed to test the hypothesis that an absence of A2BR on myeloid cells has an influence on the immunological adaptation to pregnancy and possibly influences pregnancy outcome. In a mouse model with targeted deletion of A2BR in myeloid cells (Adora2B923^f/f^‐LysM^Cre^), we observed substantial changes in myeloid and lymphoid cell populations during pregnancy. These included decreased systemic accumulation and function of MDSC, reduced numbers of uterine natural killer (uNK) cells, and altered composition of T‐cell subpopulations. However, we observed no clear effect on pregnancy outcome. Our findings suggest that the absence of A2BR on myeloid cells may influence the immunological adaptation to pregnancy, but further research is necessary to work out the clinical impact.

## Results

### Myeloid lack of A2BR leads to altered myeloid and lymphoid cell populations in spleens of pregnant mice

**Figure 1:**
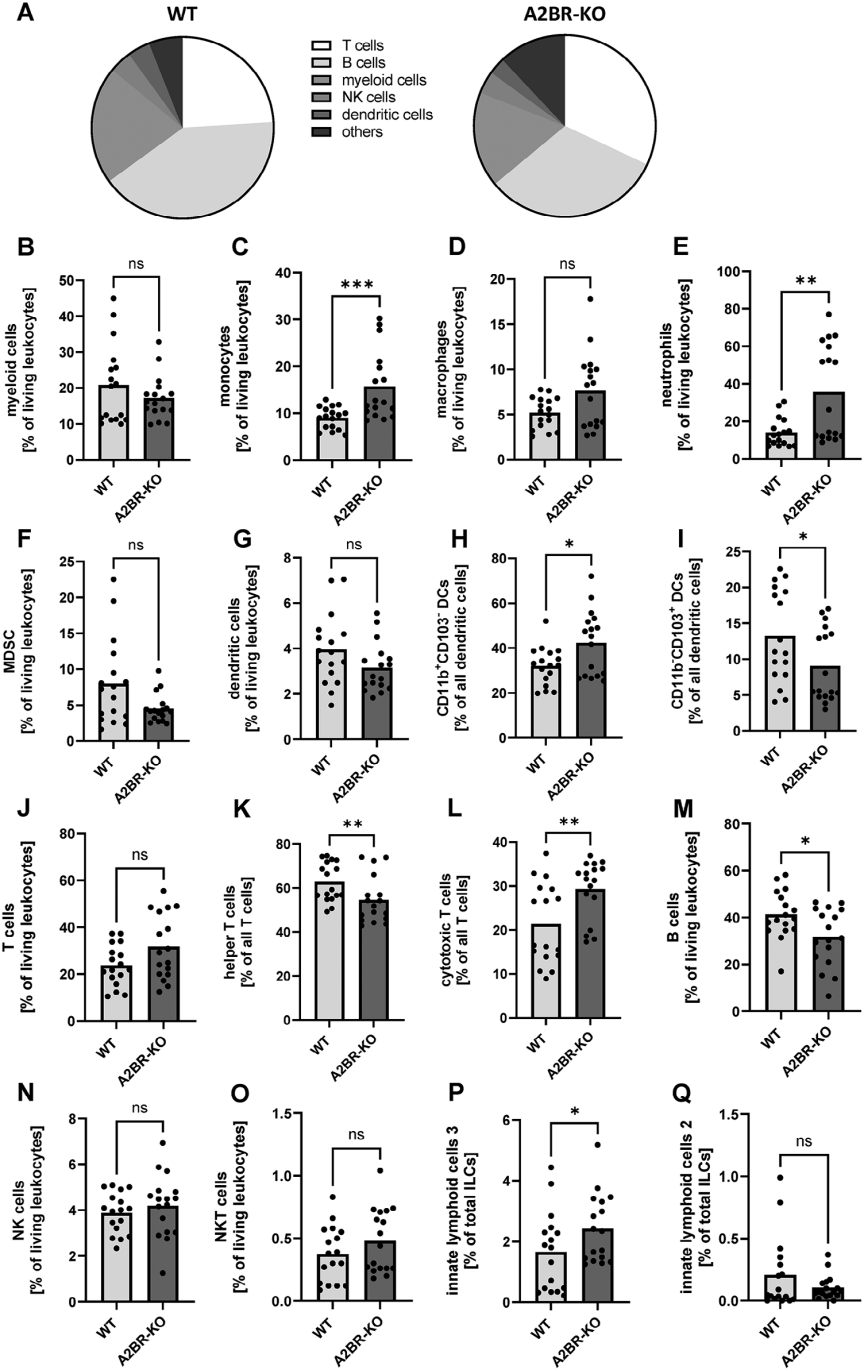
Immune cell populations in spleens of Adora2B923^f/f^‐LysM^Cre^ and wildtype mice. Wildtype (WT) and Adora2B923^f/f^‐LysM^Cre^ (A2BR‐KO) mice were time mated and the day when a vaginal plug was detected was defined as day E0.5. Mice were euthanized at E10.5, spleens were removed, homogenized, and analyzed by flow cytometry. (A) Proportions of the different immune cell populations in spleens of pregnant WT (left diagram, *n* = 17) and A2BR‐KO mice (right diagram, *n* = 17) at mid‐pregnancy (E10.5). (B–N) Percentages of monocytes (B), macrophages (C), neutrophils (D), MDSC (E), dendritic cells (F), CD11b^+^ dendritic cells (G), CD103^+^ dendritic cells (H), T cells (I), T helper cells (J), cytotoxic T cells (K), B cells (L), NK cells (M) and innate lymphoid cells 3 (N) from all spleen leukocytes in WT (*n* = 17) and A2BR‐KO mice (*n* = 17). Each symbol represents an individual animal and the mean is indicated. Light grey bars represent WT animals and dark grey bars represent A2BR‐KO animals. ***p < 0.001, **p < 0.01, *p < 0.05, ns = not significant, Mann–Whitney test.

First, we analyzed immune cell populations in spleens of C57BL/6J (WT) and Adora2B923^f/f^‐LysM^Cre^ (A2BR‐KO) mice at mid‐pregnancy (E10.5). The gating strategy for immune cell populations is shown in Supporting Information Fig. S1. Fig. 1A shows the immune cell composition of WT (left) and A2BR‐KO mice (right). The most prominent differences were observed in myeloid cell populations (Fig. 1B) with increased monocytes (15.7% ± 7.4% vs. 9.0% ± 2.4%, *n* = 17, *p* < 0.001, Fig. 1C), macrophages (7.6% ± 4.2% vs. 5.2% ± 1.7%, *n* = 17, n.s., Fig. 1D), and neutrophils (35.7% ± 25.4% vs. 14.0% ± 7.5%, *n* = 17, *p* < 0.01, Fig. 1E) but decreased MDSC (4.5% ± 2.0% vs. 8.0% ± 6.0%, *n* = 17, n.s., Fig. 1F) in A2BR‐KO mice in comparison to WT mice. The number of all dendritic cells (DC) did not differ between A2BR‐KO mice and WT mice (3.2% ± 1.1% vs. 4.0% ± 1.6%, *n* = 17, n.s., Fig. 1G) but the subpopulation of CD11b^+^/CD103^−^ DCs was increased (42.4% ± 14.4% vs. 32.0% ± 8.4%, *n* = 17, *p* < 0.05, Fig. 1H) while CD11b^−^/CD103^+^ DCs were decreased (9.1% ± 5.1% vs. 13.2% ± 6.5%, *n* = 17, *p* < 0.05, Fig. 1I). In nonpregnant animals, there were no differences observed in myeloid cell populations between WT and A2BR‐KO mice (Supporting Information Fig. S2A–F).

In lymphocytes, we found slightly increased T cells (31.8% ± 13.9% vs. 23.7% ± 8.7%, *p* = 0.0502, Fig. 1J) with decreased proportions of T helper cells (54.6% ± 10.8% vs. 62.7% ± 8.8%, *n* = 17, *p* < 0.01, Fig. 1K) and increased proportions of cytotoxic T cells (29.3% ± 6.7% vs. 21.4% ± 9.2%, *n* = 17, *p* < 0.01, Fig. 1L) in A2BR‐KO splenocytes in comparison to WT splenocytes as well as decreased B cells (31.7% ± 12.5% vs. 41.3% ± 10.2%, *n* = 17, *p* < 0.05, Fig. 1M). No differences were observed for NK and NKT cells (Fig. 1N and O). Innate lymphoid cells 3 were increased in A2BR‐KO splenocytes in comparison to WT splenocytes (2.4% ± 1.1% vs. 1.7% ± 1.3%, *n* = 17, *p* < 0.05, Fig. 1P) while innate lymphoid cells 2 did not differ (Fig. 1Q). Similar tendencies with decreased T‐helper cells were observed in nonpregnant animals, whereby no differences were observed in all T cells, cytotoxic T cells, B cells, NK cells and ILCs in nonpregnant animals (Supporting Information Fig. 2G–L).

### Myeloid lack of A2BR leads to altered T‐cell subpopulations in spleens of pregnant mice

**Figure 2:**
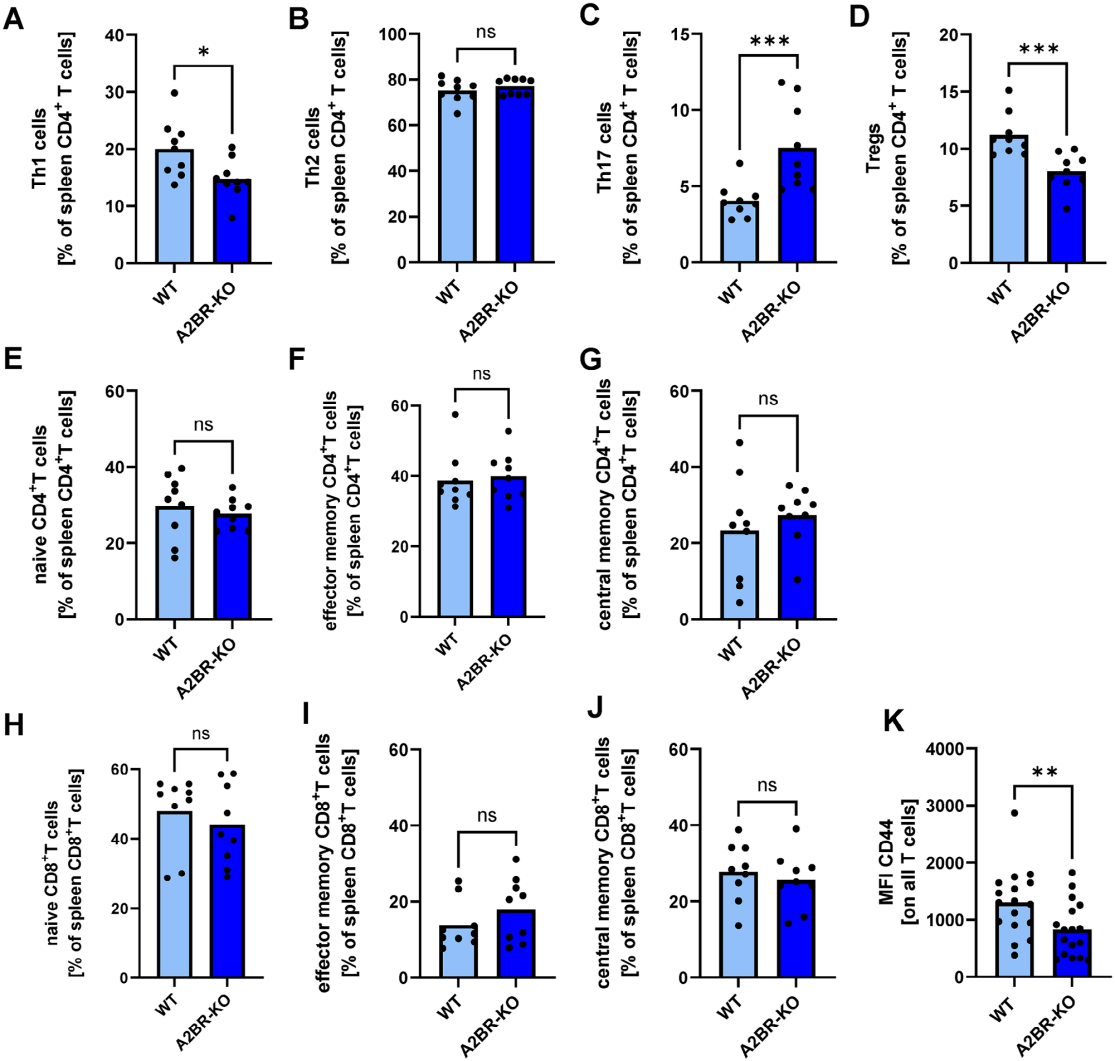
T‐cell subpopulations in spleens of Adora2B923^f/f^‐LysM^Cre^ and wildtype mice. Wildtype (WT) and Adora2B923^f/f^‐LysM^Cre^ (A2BR‐KO) mice were time mated and the day when a vaginal plug was detected was defined as day E0.5. Spleens were analyzed by flow cytometry. (A–D) Percentages of Th1 cells (A), Th2 cells (B), Th17 cells (C), and Tregs (D) from all CD4^+^ T cells, (E–J) percentages of naïve CD4^+^/CD8^+^ T cells (E, H), effector memory CD4^+^/CD8^+^ T cells (F, I) and central memory CD4^+^/CD8^+^ T cells (H, J) from all CD4^+^/CD8^+^ T cells and (K) mean fluorescence intensity of the expression of the activation marker CD44 on all T cells in WT (*n* = 9) and A2BR‐KO mice (*n* = 9). Each symbol represents an individual animal and the mean is indicated. Light blue bars represent WT animals and dark blue bars represent A2BR‐KO animals. ***p < 0.001, **p < 0.01, *p < 0.05, ns = not significant, Mann–Whitney test.

As T cells play an important role in immunologic adaptation to pregnancy, we next investigated whether there were any differences in T‐cell subpopulations between A2BR‐KO and WT splenocytes. The gating strategy for the extracellular definition of T‐cell subpopulations is depicted in Supporting Information Fig. S3A. Analysis of T‐helper cell subpopulations showed that pregnant A2BR‐KO animals had significantly fewer Th1 cells (15.0% ± 3.6% vs. 20.0% ± 5.0%, *n* = 9, *p* < 0.05, Fig. 2A) and less regulatory T cells (8.0% ± 1.6% vs. 11.2% ± 1.9%, *n* = 9, *p* < 0.001, Fig. 2D), but significantly more Th17 cells (7.5% ± 2.8% vs. 4.1% ± 1.1%, *n* = 9, *p* < .001, Fig. 2C). No differences were found in Th2 cells (Fig. 2B), naïve CD4^+^/CD8^+^ T cells (Fig. 2E and H), effector memory CD4^+^/CD8^+^ T cells (Fig. 2F and I) and central memory CD4^+^/CD8^+^ T cells (Fig. 2G and J). The expression of the activation marker CD44 on T cells was decreased in A2BR‐KO mice compared with WT mice (MFI 829 ± 474 vs. MFI 1302 ± 587, *n* = 17, *p* < 0.01, Fig. 2K). In nonpregnant animals, we already observed an increase in Th17 cells prior to pregnancy. Interestingly, percentages of Tregs were also increased in nonpregnant A2BR‐KO mice compared with WT animals, while Th1 and Th2 cells did not differ (Supporting Information Fig. S3B–E).

### Myeloid lack of A2BR leads to strongly reduced numbers of NK cells in the uteri and placentas of pregnant mice

**Figure 3:**
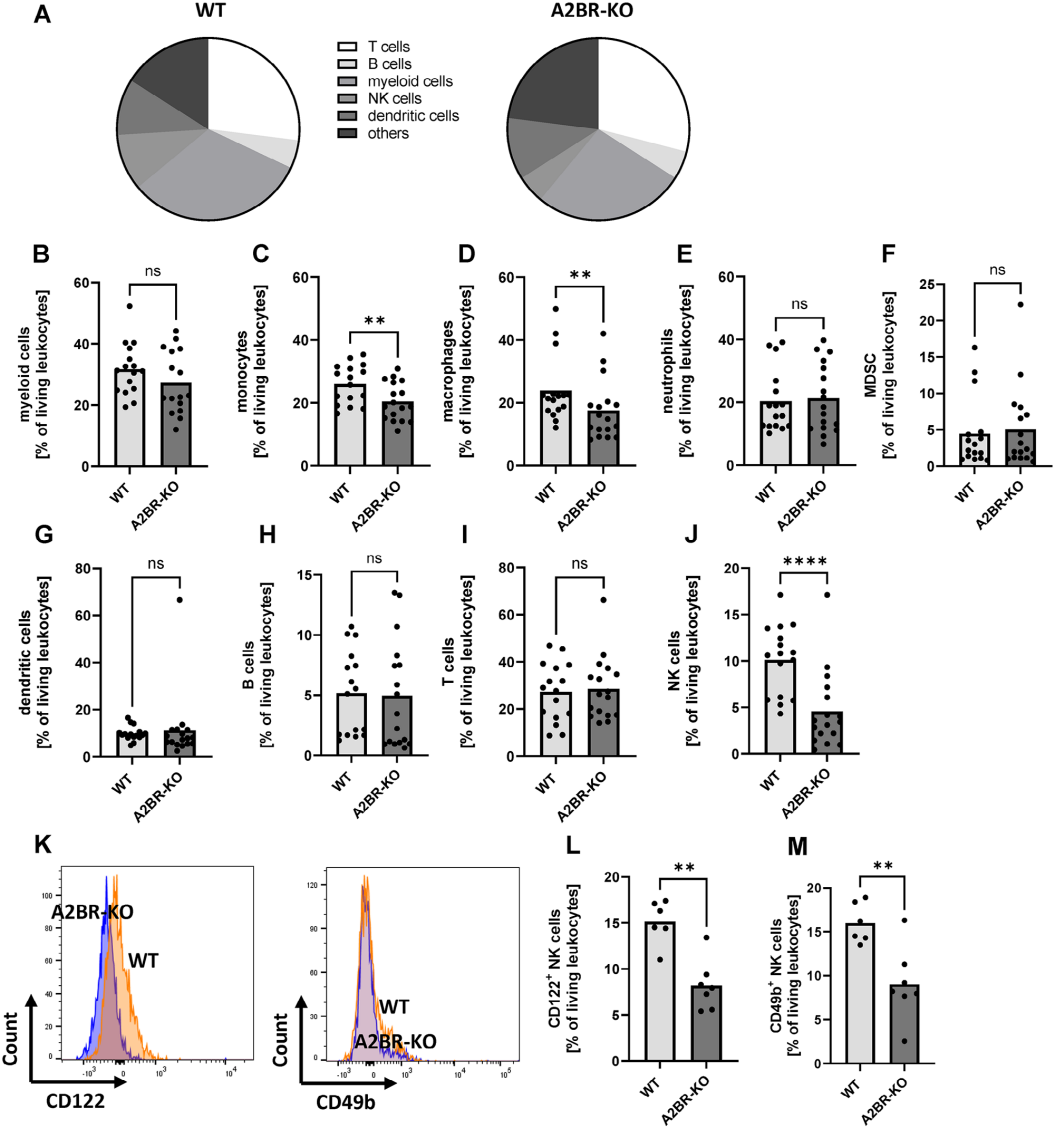
Immune cell populations in uteri of Adora2B923^f/f^‐LysM^Cre^ and wildtype mice. Wildtype (WT) and Adora2B923^f/f^‐LysM^Cre^ (A2BR‐KO) mice were time mated and the day when a vaginal plug was detected was defined as day E0.5. Mice were euthanized at E10.5, uteri were removed, homogenized, and analyzed by flow cytometry. (A) Proportions of the different immune cell populations in uteri of pregnant WT (left diagram, *n* = 17) and A2BR‐KO mice (right diagram, *n* = 17) at mid‐pregnancy (E10.5). (B–J) Percentages of myeloid cells (B), monocytes (C), macrophages (D), neutrophils (E), MDSC (F), dendritic cells (G), B cells (H), T cells (I), and NK cells (J) from all living leukocytes in uteri of WT (*n* = 17) and A2BR‐KO mice (*n* = 17). Each symbol represents an individual animal and the mean is indicated. Light grey bars represent WT animals and dark grey bars represent A2BR‐KO animals. ****p < 0.0001, **p < 0.01, ns = not significant, Mann–Whitney test. (K) Representative histogram plots showing the expression of the uNK cell markers CD122 and CD49b in uteri leukocytes of A2BR‐KO (blue) and WT mice (orange). (L, M) Bar graphs showing the proportion of CD122 expressing (L) and CD49b expressing NK cells from all NK cells in A2BR‐KO (dark grey) and WT mice (light grey). **p < 0.01, Mann–Whitney test.

The uterus is the place with the closest contact between maternal and fetal immune cells. We thus compared immune cell composition in uteri between WT and A2BR‐KO (Fig. 3A) mice at mid‐pregnancy (E10.5). In contrast to the spleen, we found decreased proportions of monocytes (20.5% ± 6.0% vs. 26.0% ± 6.2%, *n* = 16–17, *p* < 0.01, Fig. 3C) and macrophages (17.5% ± 9.5% vs. 23.9% ± 10.5%, *n* = 16–17, *p* < 0.01, Fig. 3D) in uteri of A2BR‐KO animals, but no differences in myeloid cells, dendritic cells, neutrophils or MDSC (Fig. 3B, E–G). B cells, T cells, T‐cell subpopulations, NKT cells, ILC2 cells, and ILC3 cells did also not differ between A2BR‐KO and WT mice (Fig. 3H and I; Supporting Information Fig. S4). In the uteri of nonpregnant animals, there was only a difference in the numbers of macrophages between WT and A2BR‐KO animals with increased macrophages in A2BR animals (Supporting Information Fig. S5). There was a strong reduction in NK cells in uteri of pregnant A2BR‐KO mice in comparison to WT mice (4.6% ± 4.2% vs. 10.1% ± 3.7%, *n* = 16–17, *p* < 0.0001, Fig. 3J), a difference that already existed in a weaker form before pregnancy (Supporting Information Fig. S5I). When looking at the markers for uterine NK cells ‐ CD122 and CD49b, which play a decisive role in the remodeling of uterine spiral arteries, we found that pregnant A2BR‐KO mice had significantly less uterine NK cells in comparison to WT mice (Fig. 3K). In placentas, we found a similar immune cell composition as in spleens with increased myeloid cells and monocytes, but decreased B cells, NK cells, and T helper cells as well as decreased expression of CD44 on T cells (Supporting Information Fig. S6).

### Lack of A2BR on myeloid cells leads to decreased suppressive capacity of MDSC

**Figure 4:**
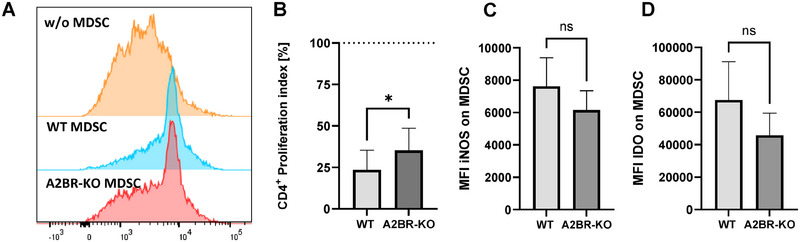
The functionality of in vitro generated MDSC from Adora2B923^f/f^‐LysM^Cre^ mice. MDSC were in vitro generated from bone marrow cells from nonpregnant wildtype (WT) and Adora2B923^f/f^‐LysM^Cre^ (A2BR‐KO) mice and added to CFSE‐stained and anti‐CD3/CD28 stimulated CD4^+^ T cells isolated from the spleens of nonpregnant mice. (A) Representative histogram plots showing proliferation of CD4^+^ T cells without (orange) and with the addition of in vitro generated MDSC from WT (blue) and (A2BR‐KO) (red) in a 2:1 ratio (T cell:MDSC). (B) Suppressive effect of in vitro generated MDSC from WT (light grey) and A2BR‐KO mice (dark grey) on T‐cell proliferation (*n* = 9). The dashed line shows the proliferation of target CD4^+^ T cells without the addition of MDSC. The proliferation index was determined as the ratio of T‐cell proliferation with and without the addition of MDSC. *p < 0.05, Wilcoxon matched‐pairs signed rank test. (C–E) Intracellular staining of effector enzymes iNOS (C) and IDO (D) on in vitro generated MDSC from WT and A2BR‐KO mice. Bars represent data from 5–9 independent experiments. Mean and standard deviation are indicated, ns = not significant, Mann–Whitney test.

Since MDSC with their suppressive activity is crucial for the maintenance of fetal–maternal tolerance during pregnancy, we investigated whether the lack of A2BR on myeloid cells may impair MDSC functionality. We found that the addition of in vitro generated MDSC isolated from A2BR‐KO mice inhibited T‐cell proliferation to a lesser extent than MDSC generated from WT mice (inhibition of T‐cell proliferation to 35.3% ± 13.4% vs. 23.5% ± 11.8%, Fig. 4A and B). Supporting Information Fig. S7A shows the concentration dependency of T‐cell suppression by MDSC from A2BR‐KO mice. Analysis of the expression of MDSC effector enzymes revealed an only slightly reduced expression of iNOS and IDO by in vitro generated MDSC from A2BR‐KO mice in comparison to in vitro generated MDSC from WT mice (iNOS: MFI 6158 ± 1190 vs. MFI 7621 ± 1767, IDO: MFI 49638 ± 15343 vs. MFI 67571 ± 23529, n.s., Fig. 4C and D). Arginase I was not expressed in in vitro generated MDSC (data not shown). The capacity of in vitro‐generated A2BR‐KO MDSC to induce Tregs in vitro did not differ from that of WT MDSC (5.6% ± 1.8% vs. 5.6% ± 1.6%, Supporting Information Fig. S7B and C).

### Lack of A2BR on myeloid cells increases proinflammatory cytokines in serum

**Figure 5:**
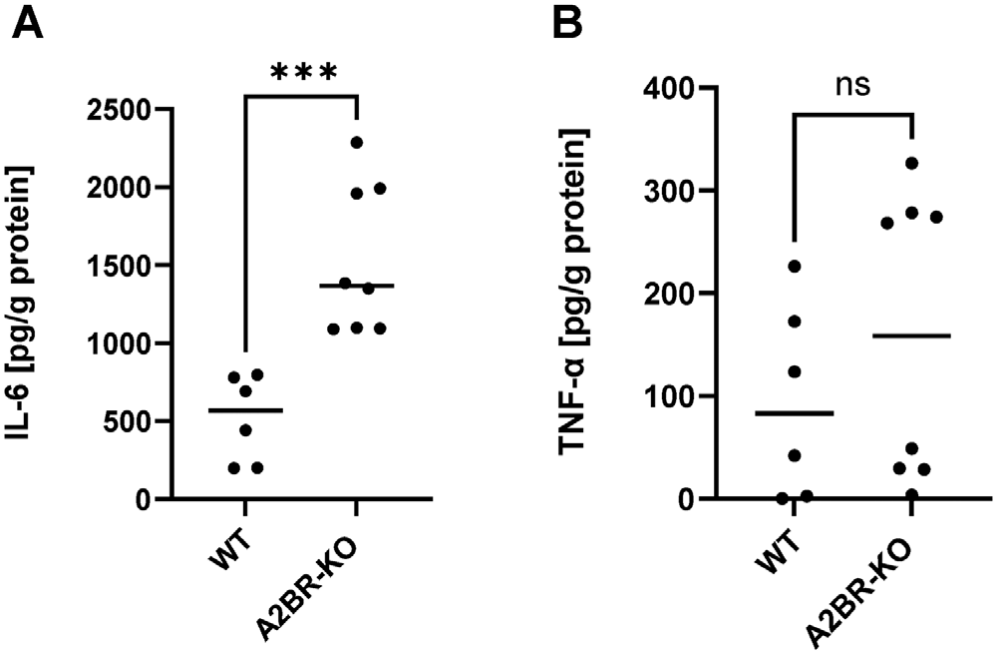
Concentrations of proinflammatory cytokines in the serum of Adora2B923^f/f^‐LysM^Cre^ and wildtype mice. Adora2B923^f/f^‐LysM^Cre^ (A2BR‐KO) and wildtype (WT) mice were time mated and the day when a vaginal plug was detected was defined as day E0.5. Mice were euthanized at day E10.5 and blood was collected. Blood was centrifuged, and serum was collected and analyzed for expression of IL‐6 and TNF‐α by ELISA. (A, B) Protein concentrations of IL‐6 (A) and TNF‐α (B) in serum of A2BR‐KO and WT mice at E10.5. Each symbol represents an individual animal and the mean is indicated (*n* = 6–8), ***p < 0.001, ns = not significant, Mann–Whitney test.

Cytokines regulate a range of biological processes throughout pregnancy. Consequently, we investigated the serum of pregnant WT and A2BR‐KO mice at day E10.5 of pregnancy, which revealed a significant elevation in proinflammatory cytokine concentrations. The protein concentration of IL‐6 in the serum of A2BR‐KO mice was found to be three times higher than that of WT mice (1532 ± 477 pg/g protein vs. 520 ± 278 pg/g protein, *n* = 6–8, *p* < 0.001, Fig. 5A). The protein concentration of TNF‐α in serum of A2BR‐KO mice was found to be marginally higher than that of WT mice (157 ± 140 pg/g protein vs. 94 ± 94 pg/g protein, Fig. 5B).

### Lack of A2BR on myeloid cells has only minor effects on the course of pregnancy

**Figure 6:**
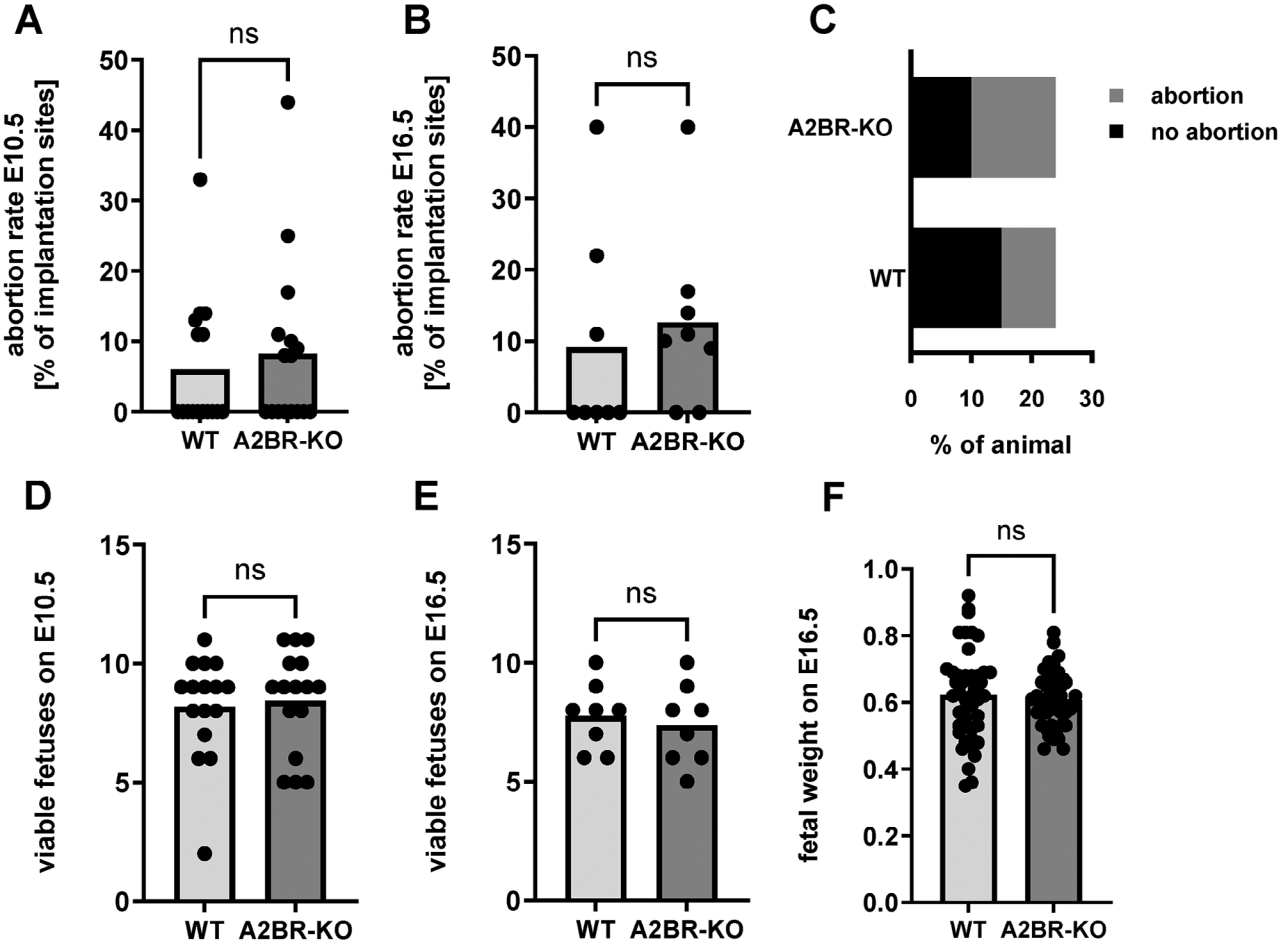
Pregnancy outcome in Adora2B923^f/f^‐LysM^Cre^ and wildtype mice. Adora2B923^f/f^‐LysM^Cre^ (A2BR‐KO) and wildtype (WT) mice were time mated and the day when a vaginal plug was detected was defined as day E0.5. Mice were euthanized at mid‐pregnancy (E10.5) or late pregnancy (E16.5) and uteri‐containing fetoplacental units were removed. Total implantation sides and resorbing units were counted and at E16.5 fetuses were weighed. (A, B) Abortion rate (percentage of resorbed fetuses per litter) of WT and A2BR‐KO mice at E10.5 (A, *n* = 16) and E16.5 (B, *n* = 8). (C) Proportion of WT and A2BR‐KO mice with and without abortions (*n* = 24). (D–F) Number of viable fetuses at E10.5 (D, *n* = 16) and E16.5 (E, *n* = 8) and weight of fetuses at E16.5 (F). Each symbol represents an individual animal and the mean is indicated. Light grey bars represent WT animals and dark grey bars represent A2BR‐KO animals. ns = not significant, Mann–Whitney test.

Finally, we investigated the influence of a lack of A2BR on myeloid cells on pregnancy outcome. Although we found significant differences in immune cell composition between pregnant A2BR‐KO mice and WT mice, there were only a few differences in pregnancy outcomes. A2BR‐KO mice had slightly but not significantly higher numbers of abortions (8.3% ± 12.1% vs. 6.0% ± 9.4%, Fig. 6A and B) with the same number of vital intrauterine fetuses at both E10.5 and E16.5 (Fig. 6D and E). When looking at the number of animals with at least one abortion, the rate was also higher in A2BR‐KO animals, but again the difference was not statistically significant (14 vs. 9 animals with abortions of a total of 24 animals, Fig. 6C). Similarly, there was no difference in the weight of the viable fetuses at E16.5 (Fig. 6F).

## Discussion

Based on findings from tumor biology, which showed that the adenosine receptor A2BR plays a role in immune tolerance to tumors [31] and that the expression of A2BR on myeloid cells seems to be involved in this process [36], we aimed to investigate the hypothesis, that a lack of A2BR on myeloid cells may also be relevant for immune tolerance during pregnancy and for pregnancy maintenance. The data presented herein demonstrate that a lack of A2BR on myeloid cells results in an imbalanced immunological adaptation during pregnancy, while there were only minor effects on pregnancy outcomes.

With regard to the quantification of immune cells in our work, an important limitation must be mentioned. We did not determine absolute cell numbers but only did a relative quantification by FACS. This is a common method and provides information about possible shifts in the immune cell populations in relation to each other [37, 38, 39]. However, quantitative effects such as an increase or decrease in all leukocytes can be overlooked.

Among myeloid cells, we observed an increase in systemic monocytes and neutrophils in pregnant A2BR‐KO mice, while macrophages and dendritic cells were unaltered and MDSC even tended to be decreased. In nonpregnant animals, there were no differences observed in myeloid cell populations between WT and A2BR‐KO mice. We assume that pregnancy‐associated factors such as adenosine promote the polarization of myeloid cells [40]. Studies described that different factors present in the plasma of pregnant women can activate monocytes. The question of which factors can activate monocytes remains to be addressed. It is suggested that these factors may be produced by the placenta [41, 42].

During a physiological pregnancy, there seems to be an increase in the number of monocytes [43] as well as activation of monocytes and a shift in monocyte subsets [44, 45]. However further activation of monocytes appears to occur in the context of inflammatory pregnancy complications such as PE [46] and elevated levels of TLR4‐expressing monocytes have been observed in patients with premature labor [47]. As for monocytes, an increase in the number of neutrophils in the peripheral blood during physiological pregnancy has been described in the literature [48]. However, a further increase and activation of neutrophils has also been observed in the context of complications such as premature labor [49, 50, 51] or preeclampsia [52, 53, 54]. Unfortunately, our study did not include any investigation of activation markers or monocyte/neutrophil functions in addition to cell numbers. Nevertheless, the elevated monocyte and neutrophil numbers observed in A2BR‐KO mice may indicate an increased immune activation, which could have a detrimental effect on pregnancy.

Our observation of reduced systemic MDSC levels during pregnancy in A2BR‐KO mice is consistent with findings from mouse tumor models showing that blockade of A2BR led to decreased MDSC accumulation and reduced tumor growth [23, 36]. Furthermore, we found that MDSC from A2BR‐KO mice exhibited a reduced capacity to suppress T‐cell proliferation in comparison to MDSC from WT animals. This is consistent with recently published results indicating that adenosine treatment significantly enhanced the immunosuppressive function of MDSC [55]. Since MDSCs play a pivotal role in pregnancy maintenance [8, 12, 13] and seem to be induced via A2BR [36], the adenosine/A2BR axis could be a potential approach to therapeutically influence pregnancy complications. Further studies are needed to evaluate this in more detail.

Interestingly, we did not only find changes in myeloid cell populations (that could be directly explained by the myeloid knockout of A2BR) but also altered lymphocyte populations during pregnancy and even before pregnancy. In addition to decreased T‐helper cells and increased cytotoxic T cells as well as decreased numbers of B cells in pregnant A2BR‐KO mice, we found altered T‐helper cell subpopulations with decreased Th1 cells and Tregs, but increased Th17 cells in pregnant A2BR‐KO animals. An altered Treg/Th17 balance has been frequently described in the context of pregnancy complications such as PE or miscarriage [56, 57]. In contrast, reduced numbers of Th1 cells are likely to be protective for pregnancy [6]. Thus, the effects of A2BR‐KO on Th1 and Th17/Treg in the context of pregnancy appear to be opposing. However, the differentiation of Th subtypes is mediated via different pathways [58], so it is quite possible that an A2BR‐KO has a different influence here. In addition, IFN‐γ, a cytokine produced by Th1 cells, inhibits the differentiation of Th17 cells and IFN‐γ‐deficient mice show significantly increased levels of Th17 cells [59], so it could be speculated that the reduced Th1 levels in pregnant A2BR‐KO mice may favor increased Th17 differentiation. In nonpregnant mice, there was a notable increase in the levels of both Th17 cells and Tregs. Typically, Th17 cells exert a proinflammatory response, whereas Tregs exert anti‐inflammatory effects on immune activation. TGF‐β is a common developmental factor shared by Th17 and Tregs, driving their differentiation in a manner that is dependent on the presence of proinflammatory cytokines such as IL‐6 and TNF‐α [60, 61]. During pregnancy TGF‐β plays a pivotal role; it is involved in the regulation of immune cell function and immune tolerance [62] but is also associated with pregnancy complications. Further functional studies of the TGF‐β‐pathway are needed to gain a deeper understanding of the Th17/Treg functions in nonpregnant mice.

With regard to cytotoxic T cells, they increase in total number during normal pregnancy [63] and are specifically regulated at the maternal‐fetal interface, where they express co‐inhibitory molecules and thus do not attack fetal cells [64]. Unfortunately, cytotoxic T cells in our study were not further phenotypically analyzed, so no statement can be made about their activation/tolerance state. Under tumor conditions, A2BR blockade led to tumor growth delay associated with increased amounts of tumor‐infiltrating CD8^+^ T cells [65]. Parallel to this, the increased numbers of cytotoxic T cells in A2BR‐KO could indicate that there is increased immune activation perhaps leading to increased risk of fetal rejection. The role of B cells in pregnancy maintenance is controversial. On the one hand, they produce protective antibodies against paternal antigens that help pregnancy to be established [66, 67], on the other hand, they can produce autoantibodies such as antiphospholipid antibodies and others that are associated with several pregnancy complications [68, 69, 70]. Taken together, the changes in the lymphocyte composition indicate a constellation in A2BR‐KO mice that is rather unfavorable for pregnancy. Further functional studies of the different cell types are needed to gain a deeper understanding of the significance of the influence of A2BR‐KO on lymphocyte homeostasis during pregnancy.

Interestingly, we found increased levels of the proinflammatory cytokines IL‐6 and TNF‐α in the serum of pregnant A2BR‐KO mice compared with pregnant WT mice. While the first phase of pregnancy — implantation and placentation — requires a local inflammatory environment [71, 72] the phase of fetal growth and development is characterized by an anti‐inflammatory milieu and ongoing proinflammatory signals or ensuing proinflammatory insults, such as infection, at this stage can lead to miscarriage and preterm birth [73, 74]. Increased levels of IL‐6 have been reported in women with recurrent pregnancy loss [75, 76] and in a murine abortion model, a reduction in the embryo resorption rate by amniotic mesenchymal stem cells was accompanied by a decrease in IL‐6 expression [77]. IL‐6 seems to be important in directing the differentiation of naïve CD4^+^ T cells onto the Th17 pathway and acts to inhibit the generation of CD4^+^ Treg cells [60], suggesting that elevated systemic IL‐6 levels are harmful to pregnancy. Conversely, IL‐6 in the uterus and placenta appears to be necessary for a successful pregnancy [78]. For TNF‐α it was shown that increased levels were associated with abortion in mice [79, 80] and there is evidence that treatment with anti‐TNF‐α antibodies may have a beneficial effect on IVF rates and improves life birth rates in women with recurrent abortions [81, 82]. Thus, our observation of increased systemic levels of proinflammatory cytokines in pregnant A2BR‐KO mice suggests that the A2BR‐KO leads to a rather unfavorable environment for pregnancy.

In the uterus, we observed a slightly different picture than in the periphery. We observed a reduction in the number of monocytes and macrophages, with a particularly notable reduction in the number of natural killer (NK) cells. Similar to our findings, Karmouty‐Quintana et al. [33] observed reduced activated alveolar macrophages in the lungs of A2BR‐KO mice following the induction of lung fibrosis by bleomycin, suggesting that A2BR plays a role in macrophage recruitment into the tissue. Decidual macrophages exhibit an anti‐inflammatory phenotype and are involved in the removal of apoptotic cells, thereby preventing proinflammatory reactions and promoting immune tolerance [83, 84, 85]. Furthermore, they play a crucial role in angiogenesis and spiral artery remodeling, which are essential processes for ensuring an adequate blood supply to the fetus and placenta [44, 86, 87]. uNK produces regulatory cytokines and growth factors, while their cytotoxic activity is significantly reduced compared with peripheral NK cells [88, 89]. Moreover, it has been proposed that they play a crucial role in trophoblast invasion into the decidua and are essential for adequate spiral artery remodeling [88, 90]. Studies in mice have demonstrated that the absence of uNK cells results in a failure of SpA remodeling between E9 and E10, as well as higher artery resistance and lower blood flow to the fetus resulting in intrauterine growth restriction, increased fetal resorption rates, PE, and miscarriage [88, 91]. The immunological effects, we observed in the uterus of A2BR‐KO mice, indicate that a myeloid knockout of A2BR may influence vascular homeostasis in the uterus and spiral artery remodeling. However, we found no fetal growth restriction in A2BR‐KO fetuses which would clinically indicate an impaired blood supply to the fetuses. Consequently, the observed immunophenotypic alterations appear to be either not functionally relevant or compensated by alternative mechanisms.

Regarding clinical effects of myeloid knockout of A2BR, we examined the rate of abortions and fetal weight in A2BR‐KO mice and control animals and found a slightly, but not statistically significant increase in the abortion rate and proportion of animals with at least one abortion in A2BR‐KO animals. Consistent with our results, mice with systemic knockout of A2BR also appear to have no relevant adverse effects on pregnancy, as reported by the commercial provider Jackson Laboratory and a scientific paper using these animals [92]. Consequently, the descriptive immunological observations could not be directly linked to a clinical pregnancy outcome. Therefore, we refrained from in‐depth mechanistic investigations, which represents a significant limitation of our work. Nevertheless, the observation of immunological alterations with impaired MDSC function and decreased systemic Treg numbers but increased Th17 numbers in pregnant A2BR‐KO mice and the tendency toward an increased abortion rate fit together and indicate that A2BR on myeloid cells seem to play a protective role for immune adaptation during pregnancy, but disruption can apparently be compensated for in other ways and thus it has only a minor clinical impact [56, 57]. At this point, it should be noted that we used a mating model in which no or only a few abortions occurred overall. It is possible that an effect of a myeloid lack of A2BR on abortions could be better identified in a murine abortion model. As previously mentioned, we also found no differences in the fetal weight between WT and A2BR‐KO animals and thus no evidence for growth retardation as a result of the myeloid A2BR knockout. Further indications of PE in the A2BR‐KO animals, which could be suspected based on the immunological effects observed in the uterus, particularly the reduced numbers of uterine NK cells, were not considered in the present study. Again, it would be beneficial to examine an animal model with a pre‐existing predisposition to PE to identify any minor effects of the myeloid A2BR knockout.

Overall, even if we were unable to show a clear correlation with a negative pregnancy outcome, our findings indicate that A2BR on myeloid cells plays a role in immune regulation during pregnancy. We assume that the absence of A2BR on myeloid cells alone may not be sufficient to cause clinically relevant effects on pregnancy outcomes. For example, the upregulation of other adenosine receptors and their downstream pathways could serve to partially compensate for the effects of A2BR absence. Possibly, A2BR could serve as a potential target for immunomodulatory approaches to improve pregnancy outcomes in case of complications. However, further studies are necessary, which in our view should focus, in particular, on the modulation of MDSC functions via A2BR and the influence of A2BR on uterine NK cells and vascularization in the uterus.

## Materials and methods

### Mice

Adora2B923^f/f^‐LysM^Cre^ (A2BR‐KO) mice used in this study were kindly provided by Prof. Holger Eltzschig (University of Texas, Health Science Center). These mice are heterozygous for the cre‐recombinase coupled to the LysM promotor expressed by myeloid cells and homozygous for loxP‐sites flanking the A2BR gene. This results in the targeted deletion of the A2BR gene in myeloid cells on the C57BL/6J background. C57BL/6J (wildtype [WT]) mice and BALB/c mice were obtained from Charles River Laboratories. All animals were maintained under pathogen‐free conditions in the research animal facility of Tuebingen University, Tuebingen, Germany. All experimental animal procedures were conducted in accordance with German federal and state regulations.

Allogeneic timed mating was performed by mating BALB/c males with A2BR‐KO or WT females aged 8–12 weeks in the afternoon. The presence of a vaginal plug the following morning was visualized to determine gestational age (E0.5 = embryonic day).

Abortion rates were determined by visually inspecting the fetal‐placental units and defined as the ratio of resorbing units to the total number of implantation sites. Resorbing units were identified as either dark, small, and necrotic or pale and small. Fetal weight was determined by weighing the E16.5 fetuses immediately after removal from the amniotic cavity.

### Tissue collection and single‐cell preparations

Mice were euthanized in their home cages by CO_2_ inhalation. Spleens and placentas were removed and homogenized using a cell strainer (Greiner bio‐one) and a syringe plunger. Red blood cells were removed by ammonium chloride lysis. Uterine horns were removed in toto. The fetuses and the fetal part of the placenta were dissected from the uteri and blood vessels were removed. Uteri were placed into PBS, cut into small pieces, and pushed through a cell strainer. All single‐cell suspensions were then adjusted to a concentration of 5 × 10^6^ cells/mL in PBS.

### Cell isolation and culture

For isolation of CD4^+^ T cells from murine splenocytes from nonpregnant WT mice, cells were labeled with T‐cell Biotin‐Antibody Cocktail followed by two consecutive Anti‐Biotin magnetic bead separation steps (Miltenyi) according to the manufacturer's instructions. The purity of CD4^+^ T cells after separation was greater than 90%, determined by flow cytometry.

In vitro generation of murine MDSC was performed according to previously established protocols [35]. For in vitro generation of MDSC, nonpregnant WT, and A2BR‐KO mice were euthanized and the femora and tibia were removed. Bone marrow was collected by rinsing the bones with PBS. Cells were washed, adjusted to 5 × 10^5^ cells/mL, and cultured for 96 h at 37°C in Dulbecco's modified eagle medium, (PanBiotech), supplemented with 10% fetal calf serum (FCS, PanBiotech) and 1% penicillin/streptomycin (P/S, PanBiotech) and 100 ng/mL recombinant murine granulocyte colony‐stimulating factor (Peprotech) as well as 12.5 ng/mL recombinant murine granulocyte‐macrophage colony‐stimulating factor (Peprotech). After 96 h, nonadherent cells were removed and washed, and adherent MDSC were detached using 0.5% trypsin/EDTA (PanBiotech).

For induction of Tregs by MDSC, in vitro generated MDSC from WT and A2BR‐KO mice were co‐cultured with freshly isolated murine CD4^+^ T cells from splenocytes of WT animals in RPMI 1640 (PanBiotech) with 10% FCS and 1% P/S in 24‐well plates at 37°C and 5% CO. After 3 days of culture, cells were harvested and intracellular Foxp3 staining was performed. CD4^+^ T cells cultured without MDSC served as control.

### T‐cell suppression assay

Freshly isolated CD4^+^ T cells from splenocytes of nonpregnant WT animals were stained with carboxyfluorescein diacetate succinimidyl ester (CFDA‐SE, Invitrogen). To inactivate CFDA‐SE, RPMI with 10% FCS was added. The number of cells was determined and the suspension was adjusted to 2 × 10^6^ cells/mL in RPMI 1640 media containing 1% P/S and 10% FCS. Labeled CD4^+^ T cells suspended in 100 µL media were stimulated with 2 × 10^5^ mouse T‐cell activator CD3/CD28 Dynabeads (Thermo Fisher Scientific) and 50 ng recombinant murine interleukin‐2 (rmIL‐2, R&D Systems) in the presence of 50 mM ß‐Mercaptoethanol (Merck). In vitro generated MDSC from WT and A2BR‐KO mice suspended in RPMI 1640 containing 1% P/S and 10% FCS and adjusted to 1 × 10^6^ cells/mL were added in different ratios. After 3 days, CD4^+^ T‐cell proliferation was determined by flow cytometry using the CFSE dye. The proliferation index, defined as the ratio of CD4^+^ T‐cell proliferation after the addition of MDSC and CD4^+^ T‐cell proliferation without MDSC, was determined. The proliferation of CD4^+^ T cells without MDSC was set to a fixed value of 1.

### Flow cytometry

For extracellular staining, freshly isolated cells were washed in FACS buffer and fluorescent‐conjugated extracellular antibodies were added. Antibodies were purchased from BD Biosciences ((CD3 FITC (145‐2C11), CD3 APC‐Cy7 (17A2), CD4 APC (RM4‐5), CD8a APC‐H7 (53‐6.7), CD11b FITC (M1/70), CD19 PE (1D3), CD45 PerCp (30‐F11), Gr1 FITC (RB6‐8C5), CD62L BV421 (MEL‐14), FSV510 Amcyan) and from BioLegend (CD4 APC‐Cy7 (GK1.5), CD25 APC (PC61), CD19 FITC (6D5), CD44 BV421 (IM7), CD11c BV421 (N418), F4/80 APC (BM8), Gr1 PE‐Cy7 (RB6‐8C5), CD103 PE (2E7), MHC II APC‐Cy7 (M5/114.15.2), Ter1.1 FITC (TER‐119), NKp46 PE‐Cy7 (29A1.4), CD122 APH‐H7 (TM‐ß1) and CD49b APC (DX5). Antibodies for intracellular staining were purchased from BD Biosciences (RORγt (Q31‐378)), BioLegend (GATA3 (16E10A23)), and Invitrogen (Foxp3 (FJK‐16s)).

For intracellular staining of Foxp3, RORγt, and GATA3, cells were extracellularly stained and then incubated in Foxp3 fixation/permeabilization working solution (Thermo Fisher Scientific). Cells were washed and stained with intracellular antibodies.

For intracellular staining of IDO and iNOS, cells were first extracellularly stained followed by incubation in BD Cytofix/Cytoperm solution (BD Bioscience), washed in BD Perm/Wash Buffer (BD Bioscience) and stained with intracellular antibodies.

For immune cell quantification, single cells were excluded and cells were pregated to living CD45^+^ cells. Among CD45^+^ cells, cell types were identified as follows: T cells CD19^−^/CD3^+^, B cells CD3^−^/CD19^+^, T helper cells CD3^+^/CD4^+^, cytotoxic T cells CD3^+^/CD8^+^, NK cells CD3^−^/NKp46^+^, NKT cells CD3^+^/NKp46^+^, neutrophils CD11b^+^/Gr1^+^, MDSC CD11b^+^/Gr1^high^, monocytes CD11b^+^/Gr‐1^−^, macrophages CD11b^+^F4/80^+^, dendritic cells CD11c^+^/MHC II^+^, innate lymphoid cells 2 Lin^−^CD3^−^/GATA3^+^ and innate lymphoid cells 3 Lin^−^/CD3^−^/NKp46^−^/RORγt^+^.

For quantification of T‐cell subpopulations, cells were pregated to CD45^+^, CD3^+^, and CD4^+^ or CD8^+^. Among CD45^+^/CD3^+^/CD4^+^ cells, cell types were identified as follows: naive T helper cells CD44^−^/CD62L^+^, effector memory T helper cells CD44^+^/CD62L^−^, central memory T helper cells CD44^+^/CD62L^+^, T helper 1 cells CXCR3^+^/CCR6^−^, T helper 2 cells CXCR3^−^/CCR6^−^, T helper 17 cells CXCR3^−^/CCR6^+^, Treg cells CD25^+^. Among CD45^+^/CD3^+^/CD8^+^ cells, cell types were identified as follows: naive cytotoxic T cells CD44^−^/CD62L^+^, effector memory cytotoxic T cells CD44^+^/CD62L^−^, central memory cytotoxic T cells CD44^+^/CD62L^+^.

Data acquisition was performed with a FACS Canto II flow cytometer (BD Bioscience) and analyzed via FlowJo V10 (FlowJo, LLC).

### Enzyme‐linked immunosorbent assay

The mouse IL‐6 and TNF‐α cytokine concentration in serum samples collected at E10.5 of pregnancy were measured using a commercial enzyme‐linked immunosorbent assay kit obtained from BioLegend according to the manufacturer's protocols. The absorbance was read at 450 nm in an iMark Microplate Reader (Bio‐Rad Laboratories). The cytokine levels were normalized to the protein concentrations of each sample. Total protein concentration was determined using the Pierce 660 nm Protein Assay Kit from Thermo Fisher Scientific according to the manufacturer´s protocols. Absorbance was measured at 660 nm using an iMark Microplate Reader (Bio‐Rad Laboratories). Protein concentration was then calculated based on the values of the standard curve.

### Statistical analysis

Statistical analysis was performed using GraphPad Prism 9.4.1 (GraphPad Software). Comparisons between two groups of unpaired and not normally distributed data were evaluated using the Mann–Whitney test. Comparisons between two groups of paired and not normally distributed data were evaluated using the Wilcoxon matched‐pairs signed rank test. A *p*‐value of less than 0.05 was considered to be statistically significant.

## Conflict of interest statement

The authors declare no conflict of interest.

## Author contributions

All authors participated in drafting or revising the article for important intellectual content and approved the final version for publication. Stefanie Dietz, Janine Hebel, Jessica Rühle, Alisha Huff, and Trim Lajqi contributed to data acquisition. Stefanie Dietz and Natascha Köstlin‐Gille analyzed and interpreted the data. Christian F. Poets, Christian Gille, and Holger K. Eltzschig provided critical feedback on intellectual content. Holger K. Eltzschig provided Adora2B923^f/f^‐LysM^Cre^ mice. Natascha Köstlin‐Gille conceived the study and Natascha Köstlin‐Gille and Stefanie Dietz wrote the paper.

## Ethics statement

All experimental animal procedures were conducted according to German federal and state regulations (approval number K04/20M).

### Peer review

The peer review history for this article is available at https://publons.com/publon/10.1002/eji.202451149

**Abbreviations**
DC: dendritic cells
FCS: fetal calf serum
MDSC: myeloid‐derived suppressor cells
NK: natural killer
P/S: penicillin/streptomycin
PE: preeclampsia
uNK: uterine natural killer

## Supporting information



Supporting information

## Data Availability

The data that support the findings of this study are available from the corresponding author upon reasonable request.

## References

[1] Rai, R. and Regan, L. , Recurrent miscarriage. Lancet 2006. 368: 601–611. 16905025 10.1016/S0140-6736(06)69204-0

[2] Goldenberg, R. L. , Culhane, J. F. , Iams, J. D. and Romero, R. , Epidemiology and causes of preterm birth. Lancet 2008. 371: 75–84. 18177778 10.1016/S0140-6736(08)60074-4 PMC7134569

[3] Stepan, H. , Kuse‐Föhl, S. , Klockenbusch, W. , Rath, W. , Schauf, B. , Walther, T. and Schlembach, D. , Diagnosis and treatment of hypertensive pregnancy disorders. Guideline of DGGG (S1‐Level, AWMF Registry No. 015/018, December 2013). Geburtshilfe Frauenheilkd 2015. 75: 900–914. 28435172 10.1055/s-0035-1557924 PMC5396549

[4] Zenclussen, A. C. , Gentile, T. , Margni, R. , Kortebani, G. and Mazzolli, A. , Asymmetric antibodies and pregnancy. Am. J. Reprod. Immunol. 2001. 45: 289–294. 11432403 10.1111/j.8755-8920.2001.450504.x

[5] Clark, D. A. , Coulam, C. B. , Daya, S. and Chaouat, G. , Unexplained sporadic and recurrent miscarrage in the new millennium: a critical analysis of immune mechanisms and treatments. Hum. Reprod. Update 2001. 7: 501–511. 11556498 10.1093/humupd/7.5.501

[6] Wegmann, T. G. , Lin, H. , Guilbert, L. and Mosmann, T. R. , Bidirectional cytokine interactions in the maternal‐fetal relationship: is successful pregnancy a TH2 phenomenon? Immunol. Today 1993. 14: 353–356. 8363725 10.1016/0167-5699(93)90235-D

[7] Moffett, A. and Loke, C. , Immunology of placentation in eutherian mammals. Nat. Rev. Immunol. 2006. 6: 584–594. 16868549 10.1038/nri1897

[8] Köstlin, N. , Hofstädter, K. , Ostermeir, A.‐L. , Spring, B. , Leiber, A. , Haen, S. , Abele, H. et al., Granulocytic myeloid‐derived suppressor cells accumulate in human placenta and polarize toward a Th2 phenotype. J. Immunol. 2016. 196: 1132–1145. 26712947 10.4049/jimmunol.1500340

[9] Dietz, S. , Schwarz, J. , Vogelmann, M. , Spring, B. , Molnár, K. , Orlikowsky, T W. , Wiese, F. et al., Cord blood granulocytic myeloid‐derived suppressor cells impair monocyte T cell stimulatory capacity and response to bacterial stimulation. Pediatr. Res. 2019. 86: 608–615. 31349362 10.1038/s41390-019-0504-7

[10] Leiber, A. , Schwarz, J. , Köstlin, N. , Spring, B. , Fehrenbach, B. , Katava, N. , Poets, C. F. et al., Neonatal myeloid derived suppressor cells show reduced apoptosis and immunosuppressive activity upon infection with *Escherichia coli* . Eur. J. Immunol. 2017. 47: 1009–1021. 28493377 10.1002/eji.201646621

[11] Rieber, N. , Gille, C. , Köstlin, N. , Schäfer, I. , Spring, B. , Ost, M. , Spieles, H. et al., Neutrophilic myeloid‐derived suppressor cells in cord blood modulate innate and adaptive immune responses. Clin. Exp. Immunol. 2013. 174: 45–52. 23701226 10.1111/cei.12143 PMC3784212

[12] Köstlin‐Gille, N. and Gille, C. , Myeloid‐derived suppressor cells in pregnancy and the neonatal period. Front. Immunol. 2020. 11: 584712. 33162999 10.3389/fimmu.2020.584712 PMC7581934

[13] Köstlin, N. , Kugel, H. , Spring, B. , Leiber, A. , Marmé, A. , Henes, M. , Rieber, N. et al., Granulocytic myeloid derived suppressor cells expand in human pregnancy and modulate T‐cell responses. Eur. J. Immunol. 2014. 44: 2582–2591. 24894988 10.1002/eji.201344200

[14] Deshmukh, H. and Way, S. S. , Immunological basis for recurrent fetal loss and pregnancy complications. Annu. Rev. Pathol. 2019. 14: 185–210. 30183507 10.1146/annurev-pathmechdis-012418-012743 PMC6566855

[15] Silva‐Vilches, C. , Ring, S. and Mahnke, K. , ATP and its metabolite adenosine as regulators of dendritic cell activity. Front. Immunol. 2018. 9: 2581. 30473700 10.3389/fimmu.2018.02581 PMC6237882

[16] Spaans, F. , De Vos, P. , Bakker, W. W. , Van Goor, H. and Faas, M. M. , Danger signals from ATP and adenosine in pregnancy and preeclampsia. Hypertension 2014. 63: 1154–1160. 24688119 10.1161/HYPERTENSIONAHA.114.03240

[17] Bours, M. J. L. , Swennen, E. L. R. , Di Virgilio, F. , Cronstein, B. N. and Dagnelie, P. C. , Adenosine 5'‐triphosphate and adenosine as endogenous signaling molecules in immunity and inflammation. Pharmacol. Ther. 2006. 112: 358–404. 16784779 10.1016/j.pharmthera.2005.04.013

[18] Okada, S. F. , Nicholas, R. A. , Kreda, S. M. , Lazarowski, E. R. and Boucher, R. C. , Physiological regulation of ATP release at the apical surface of human airway epithelia. J. Biol. Chem. 2006. 281: 22992–23002. 16754672 10.1074/jbc.M603019200 PMC2924190

[19] Roman, R. M. and Fitz, J. G. , Emerging roles of purinergic signaling in gastrointestinal epithelial secretion and hepatobiliary function. Gastroenterology 1999. 116: 964–979. 10092320 10.1016/s0016-5085(99)70081-8

[20] Burnstock, G. and Knight, G. E. , Cellular distribution and functions of P2 receptor subtypes in different systems. Int. Rev. Cytol. 2004. 240: 31–304. 15548415 10.1016/S0074-7696(04)40002-3

[21] Jacobson, K. A. and Gao, Z. G. , Adenosine receptors as therapeutic targets. Nat. Rev. Drug Discov. 2006. 5: 247–264. 16518376 10.1038/nrd1983 PMC3463109

[22] Haskó, G. , Csóka, B. , Németh, Z. H. , Vizi, E. S and Pacher, P. , A(2B) adenosine receptors in immunity and inflammation. Trends Immunol. 2009. 30: 263–270. 19427267 10.1016/j.it.2009.04.001 PMC2756472

[23] Iannone, R. , Miele, L. , Maiolino, P. , Pinto, A. and Morello, S. , Blockade of A2b adenosine receptor reduces tumor growth and immune suppression mediated by myeloid‐derived suppressor cells in a mouse model of melanoma. Neoplasia 2013. 15: 1400–IN10. 24403862 10.1593/neo.131748 PMC3884531

[24] Yang, D. , Zhang, Y. , Nguyen, H. G. , Koupenova, M. , Chauhan, A. K. , Makitalo, M. , Jones, M. et al., The A2B adenosine receptor protects against inflammation and excessive vascular adhesion. J. Clin. Invest. 2006. 116: 1913–1923. 16823489 10.1172/JCI27933 PMC1483170

[25] Ryzhov, S. , Zaynagetdinov, R. , Goldstein, A. E. , Novitskiy, S. V. , Dikov, M. M. , Blackburn, M. R. , Biaggioni, I. et al., Effect of A2B adenosine receptor gene ablation on proinflammatory adenosine signaling in mast cells. J. Immunol. 2008. 180: 7212–7220. 18490720 10.4049/jimmunol.180.11.7212 PMC3628765

[26] Mirabet, M. , Herrera, C. , Cordero, O. J. , Mallol, J. , Lluis, C. and Franco, R. , Expression of A2B adenosine receptors in human lymphocytes: their role in T cell activation. J. Cell Sci. 1999. 112 (Pt): 491–502. 9914161 10.1242/jcs.112.4.491

[27] Eckle, T. , Faigle, M. , Grenz, A. , Laucher, S. , Thompson, L. F. and Eltzschig, H. K. , A2B adenosine receptor dampens hypoxia‐induced vascular leak. Blood 2008. 111: 2024–2035. 18056839 10.1182/blood-2007-10-117044 PMC2739365

[28] Addi, A. B. , Lefort, A. , Hua, X. , Libert, F. , Communi, D. , Ledent, C. , Macours, P. et al., Modulation of murine dendritic cell function by adenine nucleotides and adenosine: involvement of the A(2B) receptor. Eur. J. Immunol. 2008. 38: 1610–1620. 18465770 10.1002/eji.200737781

[29] Novitskiy, S. V. , Ryzhov, S. , Zaynagetdinov, R. , Goldstein, A. E. , Huang, Y. , Tikhomirov, O. Y. , Blackburn, M. R. et al., Adenosine receptors in regulation of dendritic cell differentiation and function. Blood 2008. 112: 1822–1831. 18559975 10.1182/blood-2008-02-136325 PMC2518889

[30] Cekic, C. , Sag, D. , Li, Y. , Theodorescu, D. , Strieter, R. M. and Linden, J. , Adenosine A2B receptor blockade slows growth of bladder and breast tumors. J. Immunol. 2012. 188: 198–205. 22116822 10.4049/jimmunol.1101845 PMC3819109

[31] Desmet, C. J. , Gallenne, T. , Prieur, A. , Reyal, F. , Visser, N. L. , Wittner, B. S. , Smit, M. A. et al., Identification of a pharmacologically tractable Fra‐1/ADORA2B axis promoting breast cancer metastasis. Proc. Natl. Acad. Sci. U S A 2013. 110: 5139–5144. 23483055 10.1073/pnas.1222085110 PMC3612632

[32] Karmouty‐Quintana, H. , Zhong, H. , Acero, L. , Weng, T. , Melicoff, E. , West, J. D. , Hemnes, A. et al., The A2B adenosine receptor modulates pulmonary hypertension associated with interstitial lung disease. FASEB J. 2012. 26: 2546–2557. 22415303 10.1096/fj.11-200907 PMC3650483

[33] Karmouty‐Quintana, H. , Philip, K. , Acero, L. F. , Chen, N.‐Y. , Weng, T. , Molina, J. G. , Luo, F. et al., Deletion of ADORA2B from myeloid cells dampens lung fibrosis and pulmonary hypertension. FASEB J. 2015. 29: 50–60. 25318478 10.1096/fj.14-260182 PMC4763976

[34] Schingnitz, U. , Hartmann, K. , Macmanus, C. F. , Eckle, T. , Zug, S. , Colgan, S. P. and Eltzschig, H. K. , Signaling through the A2B adenosine receptor dampens endotoxin‐induced acute lung injury. J. Immunol. 2010. 184: 5271–5279. 20348420 10.4049/jimmunol.0903035 PMC2858788

[35] Dietz, S. , Schwarz, J. , Velic, A. , González‐Menéndez, I. , Quintanilla‐Martinez, L. , Casadei, N. , Marmé, A. et al., Human leucocyte antigen G and murine Qa‐2 are critical for myeloid derived suppressor cell expansion and activation and for successful pregnancy outcome. Front. Immunol. 2022. 12. 10.3389/fimmu.2021.787468 PMC8801456 35111157

[36] Ryzhov, S. , Novitskiy, S. V. , Goldstein, A. E. , Biktasova, A. , Blackburn, M. R. , Biaggioni, I. , Dikov, M. M. et al., Adenosinergic regulation of the expansion and immunosuppressive activity of CD11b+Gr1+ cells. J. Immunol. 2011. 187: 6120–6129. 22039302 10.4049/jimmunol.1101225 PMC3221925

[37] Baumer, Y. , Gutierrez‐Huerta, C. A. , Saxena, A. , Dagur, P. K. , Langerman, S. D. , Tamura, K. , Ceasar, J. N. et al., Immune cell phenotyping in low blood volumes for assessment of cardiovascular disease risk, development, and progression: a pilot study. J. Transl. Med. 2020. 18: 29. 31952533 10.1186/s12967-020-02207-0 PMC6966880

[38] Sivakumar, R. , Chan, M. , Shin, J. S. , Nishida‐Aoki, N. , Kenerson, H. L. , Elemento, O. , Beltran, H. et al., Organotypic tumor slice cultures provide a versatile platform for immuno‐oncology and drug discovery. OncoImmunology 2019. 8: e1670019. 31741771 10.1080/2162402X.2019.1670019 PMC6844320

[39] Gualdrón‐López, M. , Díaz‐Varela, M. , Toda, H. , Aparici‐Herraiz, I. , Pedró‐Cos, L. , Lauzurica, R. , Lacerda, M. V. G. et al., Multiparameter flow cytometry analysis of the human spleen applied to studies of plasma‐derived EVs from *Plasmodium vivax* patients. Front. Cell Infect. Microbiol. 2021. 11: 596104. 33732657 10.3389/fcimb.2021.596104 PMC7957050

[40] Panther, E. , Idzko, M. , Herouy, Y. , Rheinen, H. , Gebicke‐Haerter, P. J. , Mrowietz, U. , Dichmann, S. et al., Expression and function of adenosine receptors in human dendritic cells. Faseb J. 2001. 15: 1963–1970. 11532976 10.1096/fj.01-0169com

[41] Faas, M. M. , Van Pampus, M. G. , Anninga, Z. A. , Salomons, J. , Westra, I. M. , Donker, R. B. , Aarnoudse, J. G. et al., Plasma from preeclamptic women activates endothelial cells via monocyte activation in vitro. J Reprod. Immunol. 2010. 87: 28–38. 20970197 10.1016/j.jri.2010.07.005

[42] Hennessy, A. , Painter, D. M. , Orange, S. and Horvath, J. S. , Placental tissue interleukin‐10 receptor distribution in pre‐eclampsia. Am. J. Reprod. Immunol. 2003. 49: 377–381. 12951965 10.1034/j.1600-0897.2003.00053.x

[43] Sacks, G. P. , Studena, K. , Sargent, I. L. and Redman, C. W. G. , Normal pregnancy and preeclampsia both produce inflammatory changes in peripheral blood leukocytes akin to those of sepsis. Am. J. Obstet. Gynecol. 1998. 179: 80–86. 9704769 10.1016/s0002-9378(98)70254-6

[44] Faas, M. M. , Spaans, F. and De Vos, P. , Monocytes and macrophages in pregnancy and pre‐eclampsia. Front. Immunol. 2014. 5: 298. 25071761 10.3389/fimmu.2014.00298 PMC4074993

[45] Luppi, P. , Haluszczak, C. , Trucco, M. and Deloia, J. A. , Normal pregnancy is associated with peripheral leukocyte activation. Am. J. Reprod. Immunol. 2002. 47: 72–81. 11900591 10.1034/j.1600-0897.2002.1o041.x

[46] Melgert, B. N. , Spaans, F. , Borghuis, T. , Klok, P A. , Groen, B. , Bolt, A. , De Vos, P. et al., Pregnancy and preeclampsia affect monocyte subsets in humans and rats. PLoS One 2012. 7: e45229. 23028864 10.1371/journal.pone.0045229 PMC3441708

[47] Pawelczyk, E. , Nowicki, B. J. , Izban, M. G. , Pratap, S. , Sashti, N. A. , Sanderson, M. and Nowicki, S. , Spontaneous preterm labor is associated with an increase in the proinflammatory signal transducer TLR4 receptor on maternal blood monocytes. BMC Pregnancy Childbirth 2010. 10: 66. 20964862 10.1186/1471-2393-10-66 PMC2972234

[48] Chandra, S. , Tripathi, A. K. , Mishra, S. , Amzarul, M. and Vaish, A. K. , Physiological changes in hematological parameters during pregnancy. Indian J. Hematol. Blood Transfus. 2012. 28: 144–146. 23997449 10.1007/s12288-012-0175-6 PMC3422383

[49] Yuan, M. , Jordan, F. , Mcinnes, I. B. , Harnett, M. M. and Norman, J. E. , Leukocytes are primed in peripheral blood for activation during term and preterm labour†. Mol. Hum. Reprod. 2009. 15: 713–724. 19628509 10.1093/molehr/gap054 PMC2762373

[50] Vakili, S. , Torabinavid, P. , Tabrizi, R. , Shojazadeh, A. , Asadi, N. and Hessami, K. , The association of inflammatory biomarker of neutrophil‐to‐lymphocyte ratio with spontaneous preterm delivery: a systematic review and meta‐analysis. Mediat Inflamm 2021. 2021: 1. 10.1155/2021/6668381 PMC7870293 33603568

[51] Lakshmi, M. P. A. S. and Sravani, V. , Role of neutrophil‐lymphocyte ratio in determining the outcomes of preterm premature rupture of membranes. Int J. Reprod. Contraception Obstet. Gynecol. 2021. 10: 1617.

[52] Massobrio, M. , Benedetto, C. , Bertini, E. , Tetta, C. and Camussi, G. , Immune complexes in preeclampsia and normal pregnancy. Am. J. Obstet. Gynecol. 1985. 152: 578–583. 3893134 10.1016/0002-9378(85)90631-3

[53] Greer, I. A. , Haddad, N. G. , Dawes, J. , Johnstone, F. D. and Calder, A. A. , Neutrophil activation in pregnancy‐induced hypertension. BJOG: An Int. J. Obstet. Gynaecol. 1989. 96: 978–982. 10.1111/j.1471-0528.1989.tb03358.x 2775697

[54] Haeger, M. et al., Complement, neutrophil, and macrophage activation in women with severe preeclampsia and the syndrome of hemolysis, elevated liver enzymes, and low platelet count. Obstet. Gynecol. 1992. 79: 19–26. 1727579

[55] Zhou, D. , Yao, M. , Zhang, L. , Chen, Y. , He, J. , Zhang, Y. , Xu, H. et al., Adenosine alleviates necrotizing enterocolitis by enhancing the immunosuppressive function of myeloid‐derived suppressor cells in newborns. J. Immunol. 2022. 209: 401–411. 35777852 10.4049/jimmunol.2200142

[56] Aluvihare, V. R. , Kallikourdis, M. and Betz, A. G. , Regulatory T cells mediate maternal tolerance to the fetus. Nat. Immunol. 2004. 5: 266–271. 14758358 10.1038/ni1037

[57] Zenclussen, A. C. , Gerlof, K. , Zenclussen, M. L. , Sollwedel, A. , Bertoja, A. Z. , Ritter, T. , Kotsch, K. et al., Abnormal T‐cell reactivity against paternal antigens in spontaneous abortion: adoptive transfer of pregnancy‐induced CD4+CD25+ T regulatory cells prevents fetal rejection in a murine abortion model. Am. J. Pathol. 2005. 166: 811–822. 15743793 10.1016/S0002-9440(10)62302-4 PMC1602357

[58] Martinez, G. J. , Nurieva, R. I. , Yang, X. O. and Dong, C. , Regulation and function of proinflammatory TH17 Cells. Ann. New York Acad. Sci. 2008. 1143: 188–211. 19076351 10.1196/annals.1443.021 PMC5793850

[59] Harrington, L. E. , Hatton, R. D. , Mangan, P. R. , Turner, H. , Murphy, T. L. , Murphy, K. M. and Weaver, C. T. , Interleukin 17–producing CD4+ effector T cells develop via a lineage distinct from the T helper type 1 and 2 lineages. Nat. Immunol. 2005. 6: 1123–1132. 16200070 10.1038/ni1254

[60] Bettelli, E. , Carrier, Y. , Gao, W. , Korn, T. , Strom, T. B. , Oukka, M. , Weiner, H. L. et al., Reciprocal developmental pathways for the generation of pathogenic effector TH17 and regulatory T cells. Nature 2006. 441: 235–238. 16648838 10.1038/nature04753

[61] Zhou, L. , Lopes, J. E. , Chong, M. M. W. , Ivanov, I. I. , Min, R. , Victora, G. D. , Shen, Y. et al., TGF‐β‐induced Foxp3 inhibits TH17 cell differentiation by antagonizing RORγt function. Nature 2008. 453: 236–240. 18368049 10.1038/nature06878 PMC2597437

[62] Kang, X. , Zhang, X. , Liu, Z. , Xu, H. , Wang, T. , He, L. and Zhao, A. , Granulocytic myeloid‐derived suppressor cells maintain feto‐maternal tolerance by inducing Foxp3 expression in CD4+CD25‐T cells by activation of the TGF‐beta/beta‐catenin pathway. Mol. Hum. Reprod. 2016. 22: 499–511. 27016139 10.1093/molehr/gaw026

[63] Norton, M. T. , Fortner, K. A. , Oppenheimer, K. H. and Bonney, E. A. , Evidence that CD8 T‐cell homeostasis and function remain intact during murine pregnancy. Immunology 2010. 131: 426–437. 20553337 10.1111/j.1365-2567.2010.03316.x PMC2996563

[64] Saito, S. , Nakashima, A. , Shima, T. and Ito, M. , Th1/Th2/Th17 and regulatory T‐cell paradigm in pregnancy. Am. J. Reprod. Immunol. 2010. 63: 601–610. 20455873 10.1111/j.1600-0897.2010.00852.x

[65] Morello, S. and Miele, L. , Targeting the adenosine A2b receptor in the tumor microenvironment overcomes local immunosuppression by myeloid‐derived suppressor cells. Oncoimmunology 2014. 3: e27989. 25101221 10.4161/onci.27989 PMC4121336

[66] Canellada, A. , Färber, A. , Zenclussen, A. C. , Gentile, T. , Dokmetjian, J. , Keil, A. , Blois, S. et al., Interleukin regulation of asymmetric antibody synthesized by isolated placental B cells. Am. J. Reprod. Immunol. 2002. 48: 275–282. 12516641 10.1034/j.1600-0897.2002.01125.x

[67] Kelemen, K. , Bognar, I. , Paal, M. and Szekeres‐Bartho, J. , A progesterone‐induced protein increases the synthesis of asymmetric antibodies. Cell. Immunol. 1996. 167: 129–134. 8548836 10.1006/cimm.1996.0016

[68] Reece, E. A , Gabrielli, S. , Cullen, M. T. , Zheng, X.‐Z. , Hobbins, J. C. and Nigel Harris, E. , Recurrent adverse pregnancy outcome and antiphospholipid antibodies. Am. J. Obstet. Gynecol. 1990. 163(1 Pt): 162–169. 2115735 10.1016/s0002-9378(11)90692-9

[69] Ayres, M. A. and Sulak, P. J. , Pregnancy complicated by antiphospholipid antibodies. South Med. J. 1991. 84: 266–268. 1899296 10.1097/00007611-199102000-00029

[70] Muzzio, D. , Zenclussen, A. C. and Jensen, F. , The role of B cells in pregnancy: the good and the bad. Am. J. Reprod. Immunol. 2013. 69: 408–412. 23351028 10.1111/aji.12079

[71] Zenclussen, A. C. and Hämmerling, G. J. , Cellular regulation of the uterine microenvironment that enables embryo implantation. Front. Immunol. 2015. 6: 321. 26136750 10.3389/fimmu.2015.00321 PMC4470084

[72] Gnainsky, Y. , Granot, I. , Aldo, P. , Barash, A. , Or, Y. , Mor, G. and Dekel, N. , Biopsy‐induced inflammatory conditions improve endometrial receptivity: the mechanism of action. Reproduction 2015. 149: 75–85. 25349438 10.1530/REP-14-0395

[73] Mor, G. , Aldo, P. and Alvero, A. B. , The unique immunological and microbial aspects of pregnancy. Nat. Rev. Immunol. 2017. 17: 469–482. 28627518 10.1038/nri.2017.64

[74] Romero, R. , Espinoza, J. , Gonçalves, L. , Kusanovic, J. , Friel, L. and Hassan, S. , The role of inflammation and infection in preterm birth. Semin. Reprod. Med. 2007. 25: 021–039. 10.1055/s-2006-956773 PMC8324073 17205421

[75] Jameel, S. , Bhuwalka, R. , Begum, M. , Bonu, R. and Jahan, P. , Circulating levels of cytokines (IL‐6, IL‐10 and TGF‐ β) and CD4+CD25+FOXP3+Treg cell population in recurrent pregnancy loss. Reprod. Biol. 2024. 24: 100842. 38176116 10.1016/j.repbio.2023.100842

[76] Liang, P.‐Y. , Lian, R. , Xiang, L. , Shan, L. , He, K. and Wang, S. , Inflammatory shift alterations of proinflammatory and anti‐inflammatory cytokines in unexplained recurrent miscarriage patients. Reprod. Biol. 2024. 24: 100911. 38861846 10.1016/j.repbio.2024.100911

[77] Xiao, Y. , Zeng, F. and Sun, J. , The improvement of inflammatory infiltration and pregnancy outcome in mice with recurrent spontaneous abortion by human amniotic mesenchymal stem cells. Biol. Reprod. 2024. 111: 351–360. 38718142 10.1093/biolre/ioae074 PMC11327314

[78] Prins, J. R. , Gomez‐Lopez, N. and Robertson, S. A. , Interleukin‐6 in pregnancy and gestational disorders. J. Reprod. Immunol. 2012. 95: 1–14. 22819759 10.1016/j.jri.2012.05.004

[79] Arck, P. C. , Merali, F. S. , Manuel, J. , Chaouat, G. and Clark, D. A. , Stress‐triggered abortion: inhibition of protective suppression and promotion of tumor necrosis factor‐α (TNF‐α) release as a mechanism triggering resorptions in mice. Am. J. Reprod. Immunol. 1995. 33: 74–80. 7619237 10.1111/j.1600-0897.1995.tb01141.x

[80] Gelber, S. E. , Brent, E. , Redecha, P. , Perino, G. , Tomlinson, S. , Davisson, R. L. and Salmon, J. E. , Prevention of defective placentation and pregnancy loss by blocking innate immune pathways in a syngeneic model of placental insufficiency. J. Immunol. 2015. 195: 1129–1138. 26071558 10.4049/jimmunol.1402220 PMC4506873

[81] Winger, E. E. , Reed, J. L. , Ashoush, S. , Ahuja, S. , El‐Toukhy, T. and Taranissi, M. , Treatment with adalimumab (Humira) and intravenous immunoglobulin improves pregnancy rates in women undergoing IVF. Am. J. Reprod. Immunol. 2009. 61: 113–120. 19055656 10.1111/j.1600-0897.2008.00669.x

[82] Winger, E. E. and Reed, J. L. , Treatment with tumor necrosis factor inhibitors and intravenous immunoglobulin improves live birth rates in women with recurrent spontaneous abortion. Am. J. Reprod. Immunol. 2008. 60: 8–16. 18422811 10.1111/j.1600-0897.2008.00585.x

[83] Eikmans, M. , Van Der Zwan, A. , Claas, F. H. J. , Van Der Hoorn, M.‐L. and Heidt, S. , Got your mother in a whirl: the role of maternal T cells and myeloid cells in pregnancy. HLA 2020. 96: 561–579. 32841539 10.1111/tan.14055

[84] Heikkinen, J. , Möttönen, M. , Komi, J. , Alanen, A. and Lassila, O. , Phenotypic characterization of human decidual macrophages. Clin. Exp. Immunol. 2003. 131: 498–505. 12605704 10.1046/j.1365-2249.2003.02092.x PMC1808648

[85] Lidström, C. , Matthiesen, L. , Berg, G. , Sharma, S. , Ernerudh, J. and Ekerfelt, C. , Cytokine secretion patterns of NK cells and macrophages in early human pregnancy decidua and blood: implications for suppressor macrophages in decidua. Am. J. Reprod. Immunol. 2003. 50: 444–452. 14750551 10.1046/j.8755-8920.2003.00112.x

[86] Yeh, C. C. , Chao, K. C. and Huang, S. J. , Innate immunity, decidual cells, and preeclampsia. Reprod. Sci. 2013. 20: 339–353. 22814099 10.1177/1933719112450330 PMC3823393

[87] Lash, G. E. , Pitman, H. , Morgan, H. L. , Innes, B. A. , Agwu, C. N. and Bulmer, J. N. , Decidual macrophages: key regulators of vascular remodeling in human pregnancy. J. Leukoc. Biol. 2016. 100: 315–325. 26819320 10.1189/jlb.1A0815-351R

[88] Burke, S. D. , Barrette, V. F. , Gravel, J. , Carter, A. L. I. , Hatta, K. , Zhang, J. , Chen, Z. et al., Uterine NK cells, spiral artery modification and the regulation of blood pressure during mouse pregnancy. Am. J. Reprod. Immunol. 2010. 63: 472–481. 20175772 10.1111/j.1600-0897.2010.00818.x

[89] Kalkunte, S. , Chichester, C. O. , Gotsch, F. , Sentman, C. L. , Romero, R. and Sharma, S. , Evolution of non‐cytotoxic uterine natural killer cells. Am. J. Reprod. Immunol. 2008. 59: 425–432. 18405313 10.1111/j.1600-0897.2008.00595.x PMC3042548

[90] Bansal, A. S. , Joining the immunological dots in recurrent miscarriage. Am. J. Reprod. Immunol. 2010. 64: 307–315. 20528832 10.1111/j.1600-0897.2010.00864.x

[91] Cavalli, R. C. , Cerdeira, A. S. , Pernicone, E. , Korkes, H. A. , Burke, S. D. , Rajakumar, A. , Thadhani, R. I. et al., Induced human decidual NK‐like cells improve utero‐placental perfusion in mice. PLoS One 2016. 11: e0164353. 27736914 10.1371/journal.pone.0164353 PMC5063315

[92] Hua, X. , Kovarova, M. , Chason, K. D. , Nguyen, M. , Koller, B. H. and Tilley, S. L. , Enhanced mast cell activation in mice deficient in the A2b adenosine receptor. J. Exp. Med. 2007. 204: 117–128. 17200408 10.1084/jem.20061372 PMC2118413

