# Supplementary material for: Impact of the adenosine receptor A2BR expressed on myeloid cells on immune regulation during pregnancy
Source: Eur J Immunol. 2024 Oct 25;54(12):2451149. doi: 10.1002/eji.202451149 (PMC11628929; doi:10.1002/eji.202451149)
Supplement: Supplementary file 1 — Supporting information [file EJI-54-2451149-s001.pdf]

Supplementary Figure 1

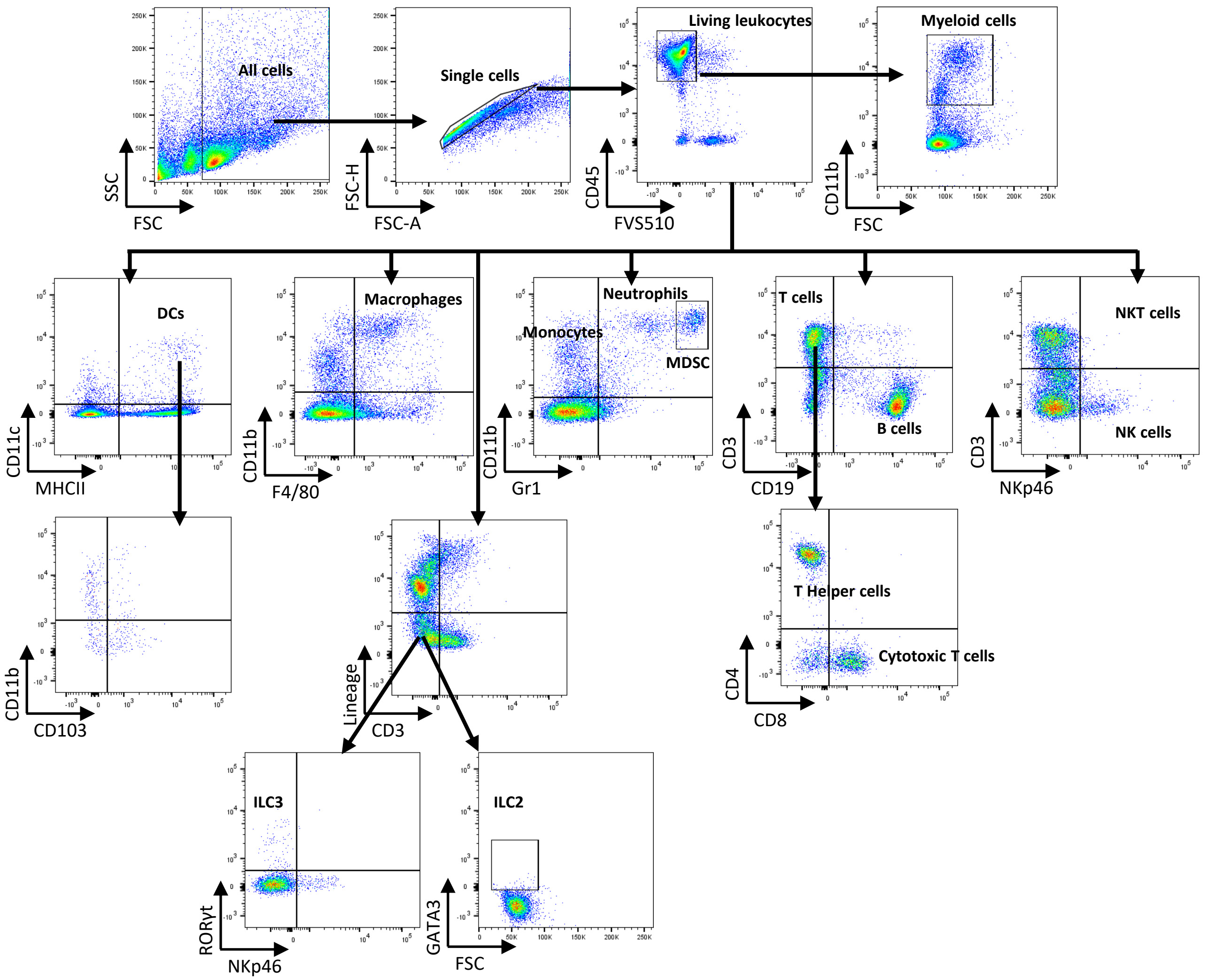

Supplementary Figure 1: Gating strategy for immune cell populations in spleens, placentas, uteri and intestines.

Representative density plots show the gating strategy for immune cell populations in spleens, placentas, uteri and intestines. Debris was excluded by gating on cells in SSC/FSC. Doublets were excluded and cells were pre-gated on living leukocytes. Within CD45^{+} leukocytes, immune cell subsets were defined as follows: myeloid cells CD11b^{+}, dendritic cells CD11b^{+}/MHC II^{+}, macrophages CD11b^{+}/F4/80^{+}, monocytes CD11b^{+}/Gr1^{-}, neutrophils CD11b^{+}/Gr1^{+}, MDSC CD11b^{+}/Gr1^{high}, T cells CD3^{+}/CD19^{-}, T Helper cells CD3^{+}/CD19^{-}/CD4^{+}, cytotoxic T cells CD3^{+}/CD19^{-}/CD8^{+}, B cells CD3^{-}/CD19^{+}, NK cells CD3^{-}/Nkp46^{+}., NKT cells CD3^{+}/Nkp46^{+}. Innate lymphoid cells (ILC) were defined as Lin^{-}CD3^{-} and for ILC3 RORyt^{+}NKp46^{-} and for IL2 GATA3^{+}.

Supplementary Figure 2

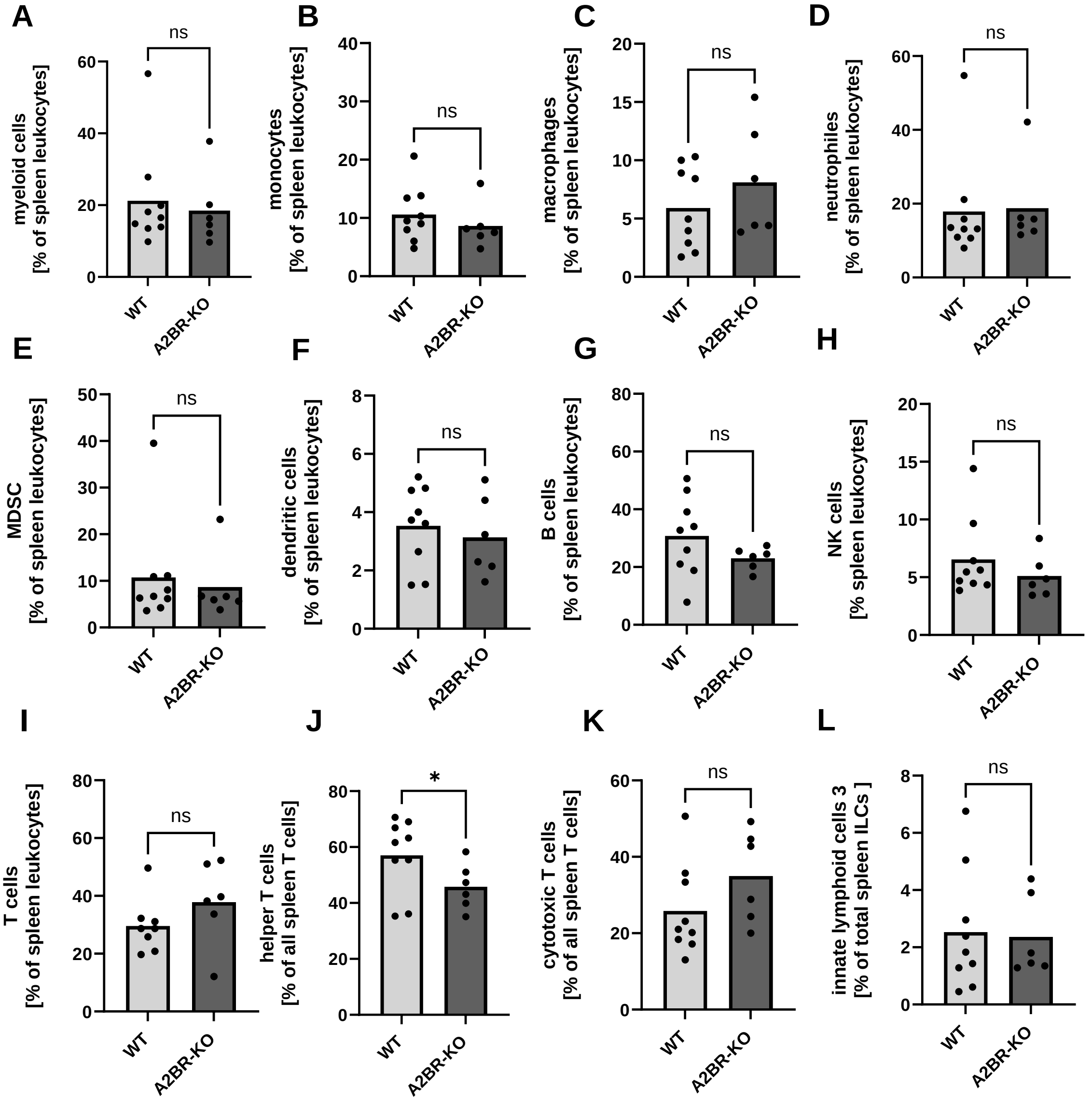

Supplementary Figure 2: Immune cell populations in spleen of non-pregnant Adora2B923$^{f/f}$-LysM^{Cre} and wildtype mice

Non-pregnant wildtype (WT) and Adora2B923$^{f/f}$-LysM^{Cre} (A2BR-KO) were euthanized and spleen were collected. Tissues were homogenized and filtered to obtain single cell suspensions and cells were analyzed by flow cytometry. (A-L) Percentages of myeloid cells (A), monocytes (B), macrophages (C), neutrophils (D), MDSC (E), dendritic cells (F), B cells (G), NK cells (H), T cells (I), T helper cells (J), cytotoxic T cells (K) and innate lymphoid cells 3 (L) from all spleen leucocytes in non-pregnant animals. Each symbol represents an individual animal and the mean is indicated (n = 6), *p<0.05, ns = not significant. Mann-Whitney test.

Supplementary Figure 3

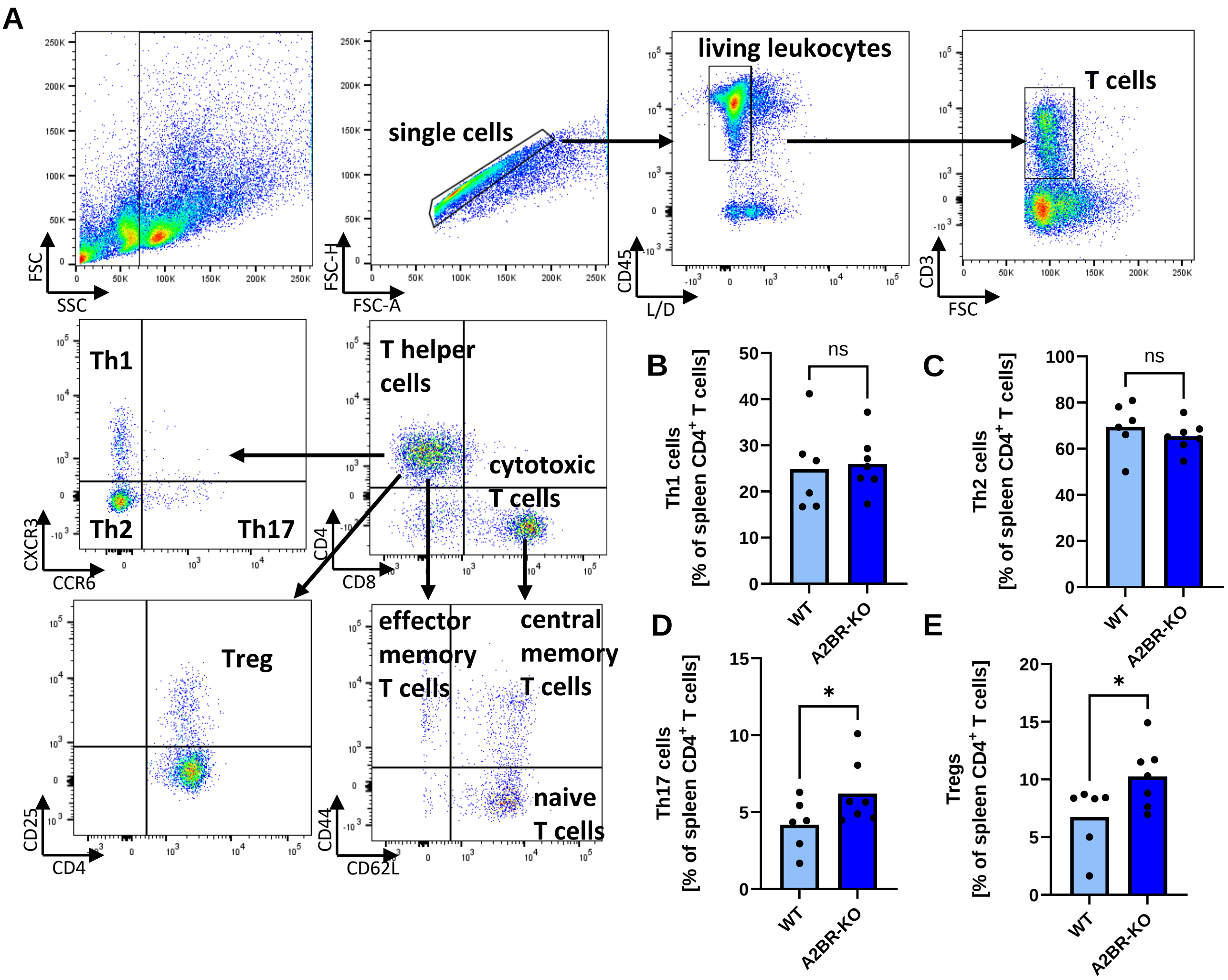

Supplementary Figure 3: T cell subpopulations in spleens and uteri

Representative density plots show the gating strategy for immune cell populations in spleens, placentas, uteri and intestines (A). Debris was excluded by gating on cells in SSC/FSC. Doublets were excluded and cells were pre-gated on living leukocytes. Within $CD3^{+}$ T cells, T cell subsets were defined as follows: T helper cells $CD4^{+}CD8^{-}$, cytotoxic T cells $CD4^{-}CD8^{+}$, Th1 T cells $CD4^{+}CXCR3^{+}CCR6^{-}$, Th2 T cells $CD4^{+}$ $CXCR3^{-}CCR6^{-}$, Th17 T cells $CD4^{+}$ $CXCR3^{-}CCR6^{+}$, Tregs $CD4^{+}CD25^{+}$, effector memory T cells $CD4^{+}/CD8^{+}CD44^{+}CD62L^{-}$, central memory T cells $CD4^{+}/CD8^{+}CD44^{+}CD62L^{+}$ and naïve T cells $CD4^{+}/CD8^{+}CD44^{-}CD62L^{+}$. (B-E) Percentages of Th1 cells (B), Th2 cells (C), Th17 cells (D) and Tregs (E) from all $CD4^{+}$ T cells in non-pregnant WT and A2BR-KO mice. Each symbol represents an individual animal and the mean is indicated. Light blue bars represent WT animals and dark blue bars represent A2BR-KO animals. *$p<0.05$, ns = not significant, Mann-Whitney test.

Supplementary Figure 4

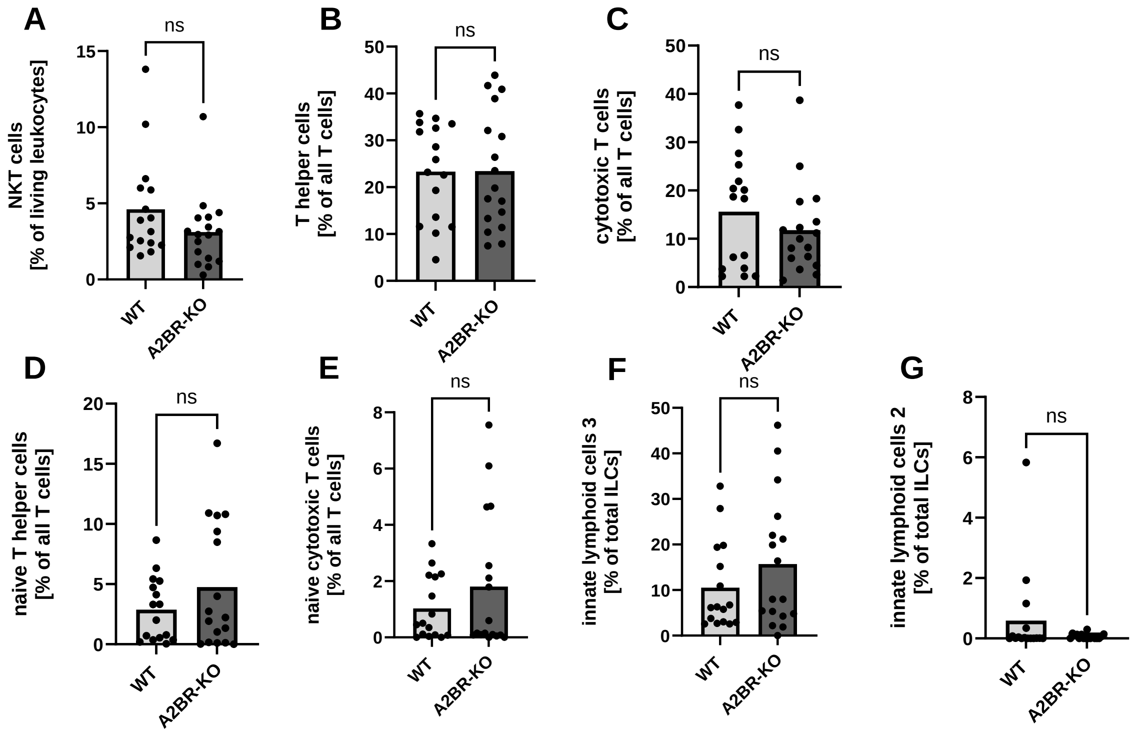

Supplementary Figure 4: Immune cell populations in uteri of pregnant Adora2B923$^{f/f}$-LysM^{Cre} and wildtype mice

Wildtype (WT) and Adora2B923$^{f/f}$-LysM^{Cre} (A2BR-KO) were time mated and the day when a vaginal plug was detected was defined as day E0.5. Mice were euthanized at E10.5 and uteri were collected. Tissues were homogenized and filtered to obtain single cell suspensions and cells were analyzed by flow cytometry. (A) Percentages of NKT cells from all spleen leucocytes in pregnant animals at E10.5. (B-G) Percentage of T helper cells (B), cytotoxic T cells (C), naïve T helper cells (D) and naïve cytotoxic T cells (E) from all splenic T cells in pregnant animals at E10.5 (F+G) Percentages of innate lymphoid cells (ILC) 2 (F) and 3 (G) from all ILCs in pregnant animals at E10.5. Light grey graphs represent WT animals and dark grey graphs represent A2BR-KO animals. Each symbol represents an individual animal and the mean is indicated (n = 17), **p<0.01, ns = not significant. Mann-Whitney test.

Supplementary Figure 5

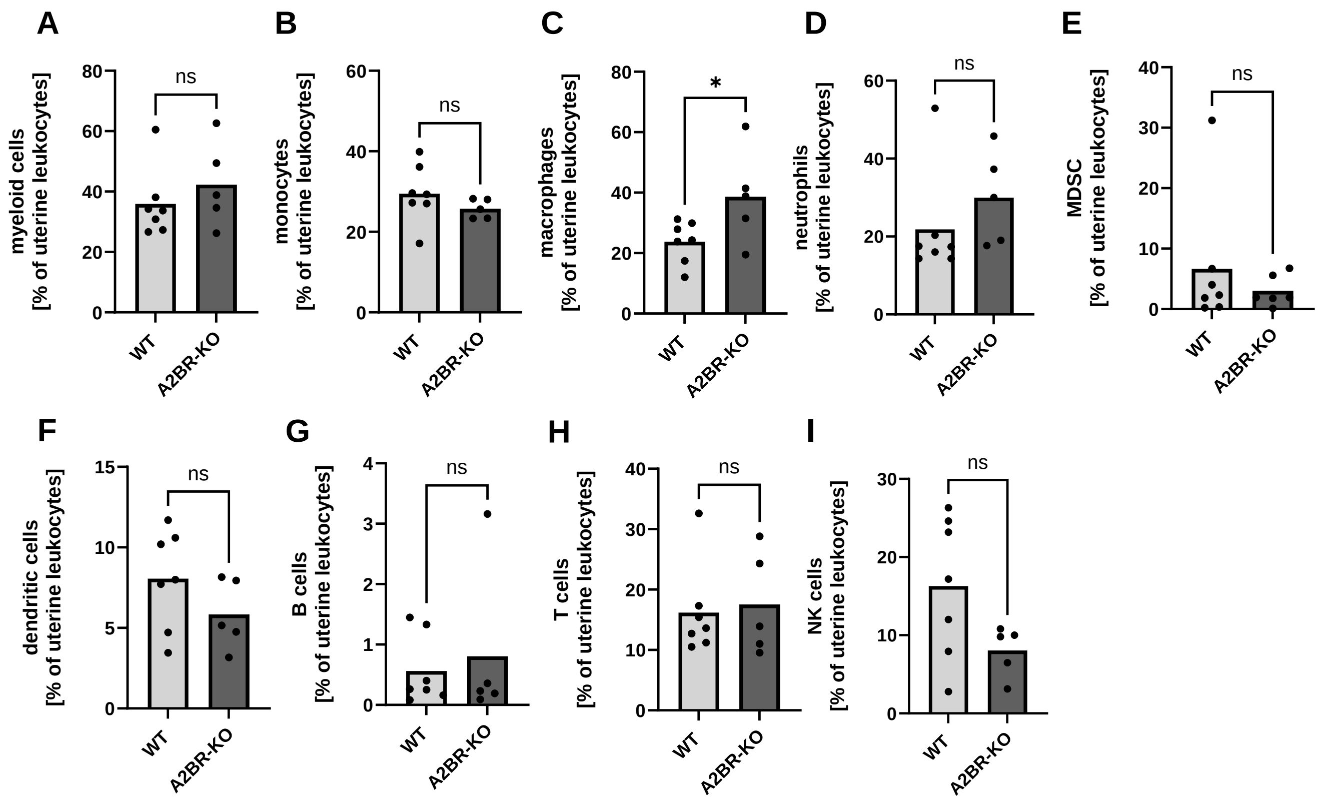

Supplementary Figure 5: Immune cell populations in uteri of non-pregnant Adora2B923$^{f/f}$-LysM^{Cre} and wildtype mice

Non-pregnant wildtype (WT) and Adora2B923$^{f/f}$-LysM^{Cre} (A2BR-KO) were euthanized and uteri were collected. Tissues were homogenized and filtered to obtain single cell suspensions and cells were analyzed by flow cytometry. (A-C) Percentages of myeloid cells (A), monocytes (B), macrophages (C), neutrophils (D), MDSC (E), dendritic cells (F), B cells (G), T cells (H) and NK cells (I) from all urine leucocytes in non-pregnant animals. Each symbol represents an individual animal and the mean is indicated (n = 6-7), *$p<0.05$, ns = not significant, Mann-Whitney test.

Supplementary Figure 6

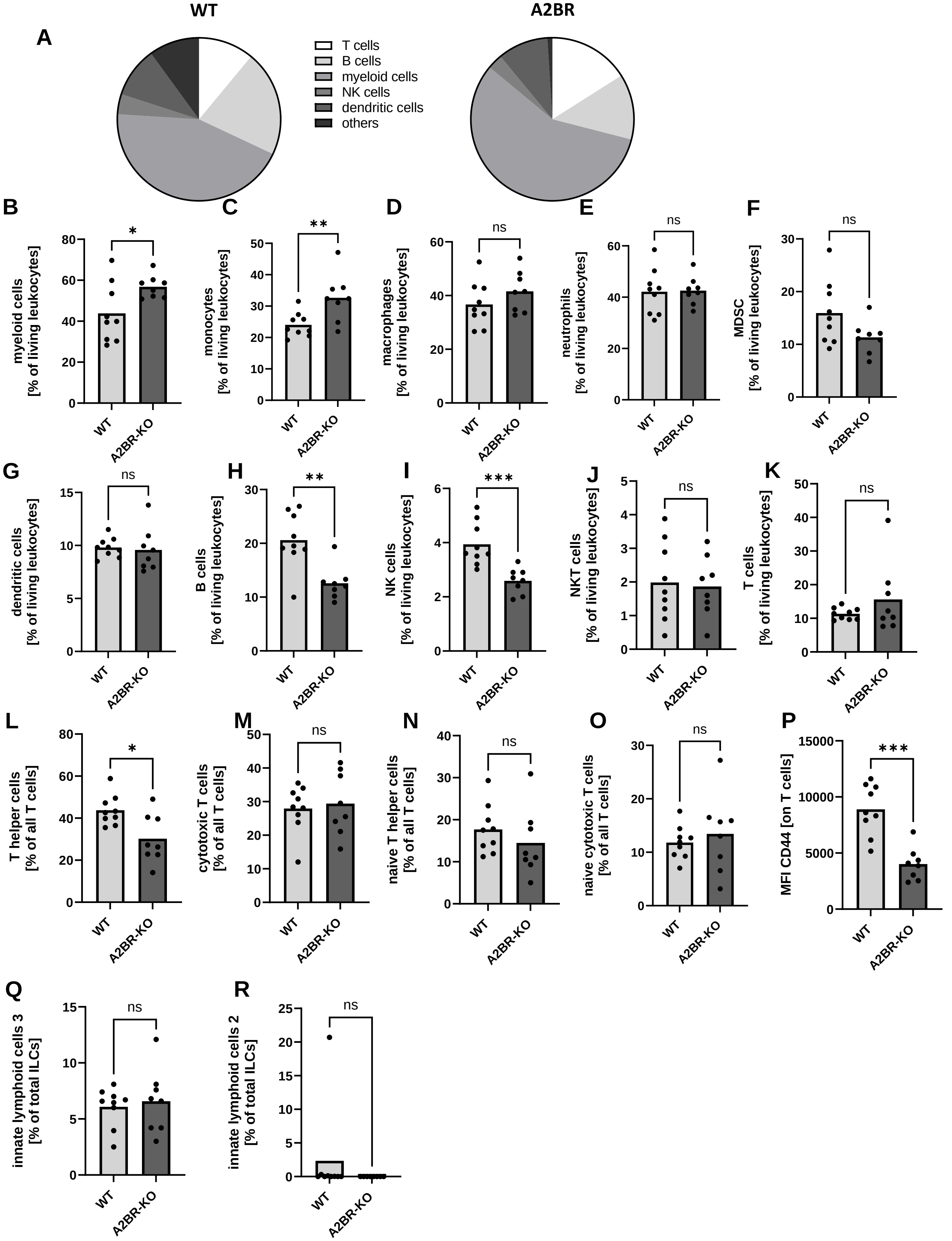

Supplementary Figure 6: Immune cell populations in placentas of pregnant Adora2B923$^{f/f}$-LysM^{Cre} and wildtype mice

Wildtype (WT) and Adora2B923$^{f/f}$-LysM^{Cre} (A2BR-KO) were time mated and the day when a vaginal plug was detected was defined as day E0.5. Mice were euthanized at E10.5 and placentas were collected. Tissues were homogenized and filtered to obtain single cell suspensions and cells were analyzed by flow cytometry. (**A**) Proportions of the different cell types in placentas of pregnant WT (left diagram, n=9) and A2BR-KO mice (right diagram, n=9) at mid-pregnancy (E10.5). **(B-S**) Percentages of myeloid cells (**B**), monocytes (**C**), macrophages (**D**), neutrophils (**E**), MDSC (**F**), dendritic cells (**G**), B cells (**H**), NK cells (**I**), NKT cells (**J**), T cells (**K**), T helper cells (**L**), cytotoxic T cells (**M**), naïve T helper cells (**N**) and naïve cytotoxic T cells (**O**) from all living leukocytes in uteri of WT (n=9) and A2BR-KO mice (n=8). (**P**) Mean Fluorescence Intensity (MFI) of CD44 on T cells. (Q+**R**) Percentages of ILC3 (**Q**) and ILC2 (**R**) from all innate lymphoid cells. Each symbol represents an individual animal and the mean is indicated. Light grey bars represent WT animals and dark grey bars represent A2BR-KO animals. ***p<0.001, **p<0.01, *p<0.05, ns = not significant, Mann-Whitney test.

Supplementary Figure 7

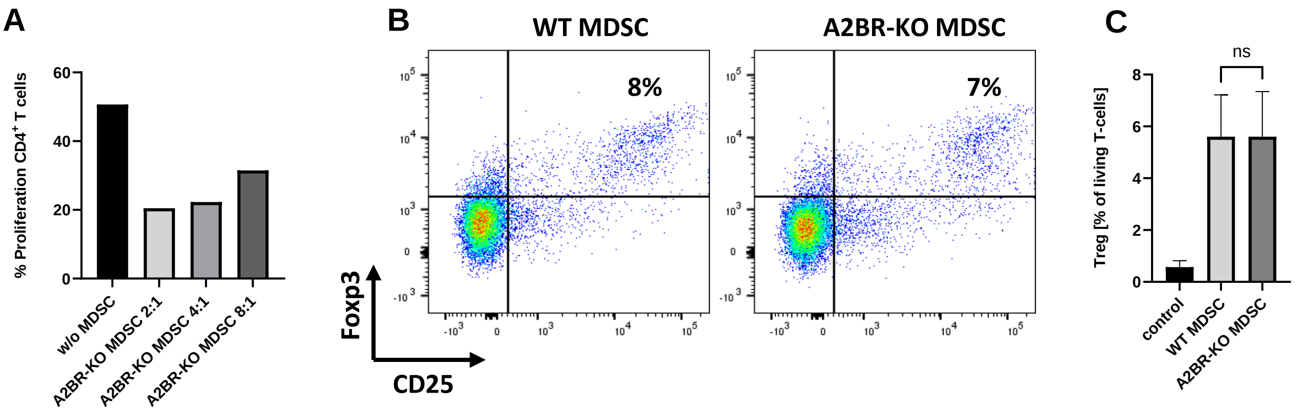

Supplementary Figure 7: Induction of regulatory T cells of *in-vitro* generated MDSC from Adora2B923$^{f/f}$-LysM^{Cre} and wildtype mice

MDSC were *in-vitro* generated from bone marrow cells from non-pregnant wildtype (WT) and Adora2B923$^{f/f}$-LysM^{Cre} (A2BR-KO) mice. (**A**) Concentration dependent suppressive T cell activity of WT and A2BR MDSC. (**B**) Representative pseudocolor plots for Foxp3 versus CD25 showing the population of Foxp3^{+} Treg cells after four days of co-culture with in-vitro generated MDSC from WT and A2BR-KO mice in a 2:1 ratio (T cells:MDSC) in the upper right quadrant. Cells were pre-gated on CD45^{+}, CD3^{+} and CD4^{+}. (**C**) Percentages of Foxp3^{+} Treg cells of all living T cells without addition of MDSC (black bar), with addition of MDSC generated from WT mice (light grey) and with addition of MDSC generated from A2BR-KO mice (dark grey, n = 6), ns = not significant, Wilcoxon matched-pairs signed rank test.
